# Recommendations for the surgical treatment of endometriosis Part 2: deep endometriosis †‡¶

**Published:** 2020-03-27

**Authors:** Working group of ESGE, ESHRE and WES, Joerg Keckstein, Christian M Becker, Michel Canis, Anis Feki, Grigoris F Grimbizis, Lone Hummelshoj, Michelle Nisolle, Horace Roman, Ertan Saridogan, Vasilios Tanos, Carla Tomassetti, Uwe A Ulrich, Nathalie Vermeulen, Rudy Leon De Wilde

**Affiliations:** Endometriosis Centre Dres. Keckstein, Richard-Wagner Strasse 18, 9500 Villach, Austria;; Nuffield Department of Obstetrics and Gynaecology, University of Oxford, John Radcliffe HospitalWomens Centre, OX3 9DU Oxford, UK;; Department of Gynaecological Surgery, University Clermont Auvergne CHU, Estaing 1 Place Lucie Aubrac, 63000 Clermont-Ferrand, France;; Department of Obstetrics and Gynecology, HFR Fribourg Hopital cantonal, 1708 Fribourg, Switzerland;; 1st Department of Obstetrics and Gynecology, Medical School Aristotle University of Thessaloniki, Tsimiski 51 Street, 54623 Thessaloniki, Greece;; World Endometriosis Society, London N1 3JS, UK;; Hôpital de la Citadelle, Department of Obstetrics & Gynecology, 4000 Liège, Belgium;; Endometriosis Centre, Clinic Tivoli-Ducos, Bordeaux, France;; Department of Obstetrics and Gynaecology, Aarhus University Hospital, Aarhus, Denmark;; Reproductive Medicine Unit, Elizabeth Garrett AndersonWing Institute for Women’s Health, University College Hospital, NW1 2BU London, UK;; Department of Obstetrics and Gynecology, Aretaeio Hospital, 2024 Nicosia, Cyprus;; Department of Obstetrics and Gynaecology, Leuven University Fertility Centre, University Hospital Leuven, 3000 Leuven, Belgium;; Department of Obstetrics and Gynaecology, Martin Luther Hospital, 14193 Berlin, Germany;; ESHRE, Central office - Meerstraat 60, BE 1852 Grimbergen, Belgium;; University Hospital for Gynecology, Carl von Ossietzky Universitat Oldenburg, 26129 Oldenburg, Germany.

**Keywords:** endometriosis, laparoscopy, surgery, deep endometriosis, extrapelvic, frozen pelvis, hysterectomy, good practice recommendations

## Abstract

**Study question:**

How should surgery for endometriosis be performed?

**Summary answer:**

This document provides recommendations covering technical aspects of different methods of surgery for deep endometriosis in women of reproductive age.

**What is known already:**

Endometriosis is highly prevalent and often associated with severe symptoms. Yet compared to equally prevalent conditions it is poorly understood and a challenge to manage. Previously published guidelines have provided recommendations for (surgical) treatment of deep endometriosis, based on the best available evidence, but without technical information and details on how to best perform such treatment in order to be effective and safe.

**Study, design, size, duration:**

A working group of the European Society for Gynaecological Endoscopy (ESGE), European Society of Human Reproduction and Embryology (ESHRE) and the World Endometriosis Society (WES) collaborated on writing recommendations on the practical aspects of surgery for treatment of deep endometriosis.

**Participants, materials, setting, methods:**

This document focused on surgery for deep endometriosis, and is complementary to a previous document in this series focusing on endometrioma surgery.

**Main results and the role of chance:**

The document presents general recommendations for surgery for deep endometriosis, starting from preoperative assessments and first steps of surgery. Different approaches for surgical treatment are discussed and are respective of location and extent of disease; uterosacral ligaments and rectovaginal septum with or without involvement of the rectum, urinary tract or extrapelvic endometriosis. In addition, recommendations are provided on the treatment of frozen pelvis and on hysterectomy as a treatment for deep endometriosis.

**Limitations, reasons for caution:**

Owing to the limited evidence available, recommendations are mostly based on clinical expertise. Where available, references of relevant studies were added.

**Wider implications of the findings:**

These recommendations complement previous guidelines on management of endometriosis and the recommendations for surgical treatment of ovarian endometrioma.

**Study funding - Competing interest(s):**

The meetings of the working group were funded by ESGE, ESHRE and WES.

Dr. Roman reports personal fees from ETHICON, PLASMASURGICAL, OLYMPUS, and NORDIC PHARMA, outside the submitted work; Dr. Becker reports grants from Bayer AG, Volition Rx, MDNA Life Sciences, and Roche Diagnostics Inc, and other relationships or activities from AbbVie Inc, and Myriad Inc, during the conduct of the study; Dr. Tomassetti reports non-financial support from ESHRE, during the conduct of the study; non-financial support and other from Lumenis, Gedeon-Richter, Ferring Pharmaceuticals, and Merck SA, outside the submitted work. The other authors had nothing to disclose.

## What does this mean for patients?

This paper was produced by a European working group looking at the different types of surgery for endometriosis, a common condition where tissue, which is similar to the lining of the womb, is found elsewhere in the body. The working group looked specifically at how best to treat a type of the disease, named deep endometriosis. Drug therapies may be used to treat endometriosis, but when endometriomas are found and need treatment, surgery is often used. There are risks associated with surgery, and especially repeated surgery, including adhesions.

The working group looked at the main types of surgery which are used to treat deep endometriosis, focussing on the different locations in the pelvis where the lesions can be found. The paper discusses in detail how different types of surgery should be performed, taking potential risks into consideration, and stresses that careful planning and involving different surgeons specialising in bowel or bladder is essential to ensure the best outcomes.

## Introduction

Endometriosis is highly prevalent, yet compared to equally prevalent conditions it is poorly understood and a challenge to manage. It has been estimated that more than 176 million women worldwide suffer from endometriosis and its associated symptoms including infertility, cyclical and non-cyclical abdominal pain, dysmenorrhea, dyspareunia, dysuria, and dyschezia (Adamson et al., 2010). It is generally accepted that no correlation exists between the severity of such pain symptoms and the extent of disease as characterised by the most commonly used revised American Fertility Society/American Society for Reproductive Medicine (rASRM) staging system (Vercellini et al., 2007), however with the use of other classification systems, such as ENZIAN, a correlation with pain symptoms may exist (Montanari et al., 2019). It has been shown that women with surgically verified endometriosis reported the highest pain symptoms compared to women with other gynaecologic pathology (Schliep et al., 2015). However, the correlation between location and depth of endometriotic lesions and pain location is poor (Hsu et al., 2011). Therefore it could be relevant to include histology when classifying the disease (Bouquet de Joliniere et al., 2019).

Endometriosis may be categorized into three entities: peritoneal endometriosis, ovarian endometriotic cysts (endometrioma), and deep endometriosis (DE) (previously known as deep infiltrating endometriosis or DIE) (Nisolle and Donnez, 1997). In addition to medical therapy with hormones and analgesics, surgery has been shown to significantly improve endometriosis-associated symptoms (Duffy et al., 2014; Byrne et al., 2018). However, like medical intervention, surgery is not always successful and is also associated with clinically relevant risks (Chapron et al., 1998; Becker et al., 2017). Treatment failure can be partially attributed to the heterogeneity of endometriosis and, in the case of surgical intervention, is directly correlated with factors such as surgical experience, the complexity of each case, and anatomical locations of the disease.

A working group comprising members of the European Society for Gynaecological Endoscopy (ESGE), European Sociaty of Human Reproduction and Embryology (ESHRE), and the World Endometriosis Society (WES) has set out to produce a series of recommendations on the practical aspects of endometriosis surgery. The first part of this series on endometrioma surgery was published in 2017 (Working group of ESGE-ESHRE and WES et al., 2017). This second part focuses on the surgical management of DE. After general considerations, such as definition and anatomical specifications, this publication concentrates on recommendations related to pre-operative management and surgical technique respective of location and extent of disease. Choices of different treatment options for DE and the selection of patients that would benefit from surgery are beyond the scope of this document. Conservative treatment, including pain management, has to be considered thoroughly.

## Materials and methods

Previously published guidelines have provided recommendations on the management of endometriosis, based on the best available evidence (Johnson et al., 2013; Dunselman et al., 2014; Ulrich et al., 2014). However, these guidelines were not intended to provide recommendations on the technical details of surgical procedures.

Due to the scarcity of evidence on these technical details, the current recommendations are primarily based on expert opinion on best clinical practice. Studies and trials of these approaches have been cited in the text, when available.

In addition to the recommendations, the working group has set up a web platform with videos on the different options available for surgery of DE. The web platform is accessible through the following link (https://www.eshre.eu/surendo) or via the ESGE, ESHRE or WES websites.

## (Results) Good Practice Recommendations

### Deep Endometriosis

#### Definition of DE

Rokitansky was probably the first to describe DE in the uterus (Rokitansky C., 1860; Hudelist et al., 2009; Tsui et al., 2015). Thomas Cullen later described DE, although he described it as adenomyosis of the round ligament (Cullen, 1896). In modern times, using laser excision, Martin et al. described in a landmark study that the lesions in approximately one-third of the women were penetrating more than 4 mm under the peritoneal surface (Martin et al., 1989). Cornillie et al. then investigated the activity of endometriosis tissue at different sub-peritoneal depths and suggested that DE was invasive (Cornillie et al., 1990), however clear evidence of infiltration is still missing. Histologically, the investigators found active glandular and stromal tissue as defined by the presence of mitoses and glycogen accumulation 5 mm below the peritoneal surface. Fibromuscular hyperplasia, cystic transformation of the glands and perivascular mononuclear inflammatory cells were noted. Other definitions of DE have been used related to the location of the disease including the involvement of bowel, bladder, ureter, vagina, parametrium (cardinal ligament), and diaphragm (Keckstein, 2017). Another definition by Bazot described DE as a fibrous/muscular infiltration of organs and anatomical structures containing endometrial tissue below the peritoneum, regardless of the depth of infiltration (Bazot and Darai, 2017).

For the purpose of this publication, and the surgical treatment of the disease, the working group is defining DE as the involvement of endometrial- like tissue with a depth of more than 5mm (Koninckx et al., 2012).

#### Morphological considerations

Infiltration of the abdominal and pelvic parietal peritoneum by endometriotic tissue can lead to involvement of retroperitoneal structures depending on the location and depth of the endometriotic nodule. DE is often associated with fibrotic changes. Thus, retraction of surrounding structures is common, and this is to be considered during pre-operative and intra-operative planning and assessment of the optimal surgical approach.

Surgeons must have a significant knowledge of pelvic anatomy in order to have an approach to a grossly distorted surgical field. Thus, pelvic anatomical landmarks represent essential points of reference to start procedures such as mobilization of the pelvic viscera, wide peritoneal resections, or the identification of further anatomical structures to be preserved, such as bowel, ureter, vessels, and parasympathetic and orthosympathetic pelvic neural fibres in nerve-sparing procedures (Ceccaroni et al., 2018). The preparation or dissection of specific anatomical spaces (Latzko, Okabayashi, Yabuki), which have been described by various authors, helps to identify these landmarks in order to facilitate a complete and safe excision of the deep lesions (Yabuki et al., 2005; Ceccaroni et al., 2018; Hudelist et al., 2018; Puntambekar and Manchanda, 2018).

The abdominal (upper third) part of the retroperitoneally located ureter follows the ventral side of the psoas muscle. Entering the pelvis, the ureter crosses dorsally to the ovarian artery and vein, and then ventrally to either the common (left side) or external (right side) iliac artery. It then courses ventrally directly below the peritoneum of the pelvic sidewall and runs antero-laterally from the uterosacral ligament. Before entering the urinary bladder, the ureter crosses the uterine artery caudally. Ureteral blood supply originates from the renal artery (upper third), ovarian artery, and abdominal aorta (middle third) as well as the internal iliac arteries and smaller vessels (lower third) and is usually located at the lateral side of the ureter. Plenty of anastomoses of these blood vessels exist within the ureteral adventitia where also the ureteral nerve plexus can be found. Anatomic variations are common, for example the prevalence of a duplex ureter has been described in 0.5% – 6% of cases and has to be taken into consideration when removing or ablating endometriotic lesions or nodules in the pelvis (Phillips et al., 1987). In addition, the normal anatomical position of the ureter can be altered significantly in the presence of DE, such as medial displacement due to surrounding fibrosis and/or infiltration. Ureteral stricture or complete obstruction may lead to hydro-ureter and hydronephrosis (this is further discussed in the section on urinary tract endometriosis).

The bilateral inferior hypogastric plexus supplies the pelvic organs, such as the rectum, bladder, and cervix, with sympathetic and parasympathetic nerve fibres and receives visceral afferent fibres. The sympathetic nerve fibres come from the superior hypogastric plexus (L3-L4) via the hypogastric nerve, which follows the common and the internal iliac artery: they are found in the pararectal space medial and dorsal to the ureter. The parasympathetic fibres originate from the pelvic and sacral splanchnic nerves (S2-S4).

**Figure 1:**
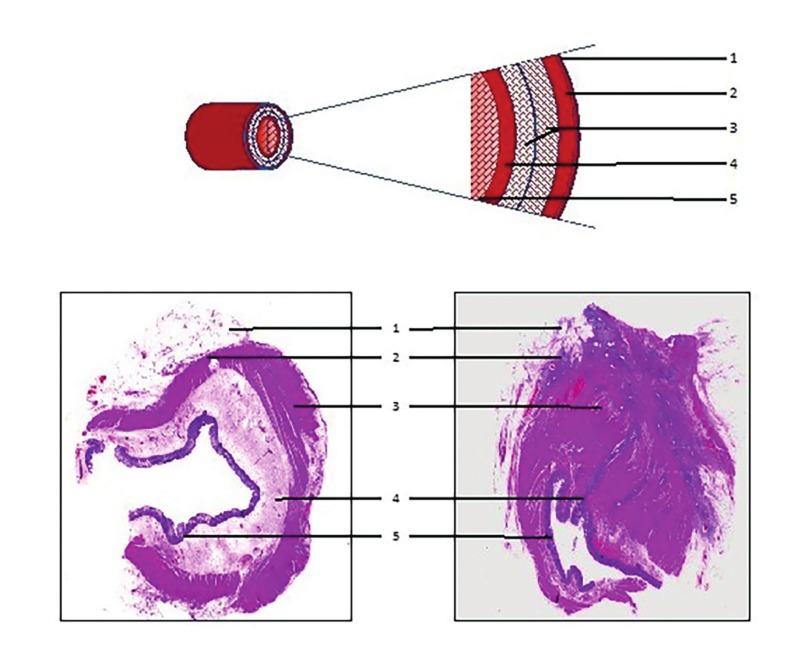
The layers of the recto-sigmoid colon, as graphical representation (upper figure), in eosin-stained healthy recto-sigmoid colon (lower left) and eosin-stained endometriosis-affected recto-sigmoid colon (lower right). 1. Serosa (sigmoid) or Adventitia (rectum); 2. Subserosa; 3. Tunica muscularis (outer longitudinal layer, inner circular layer) with Plexus myentericus in between; 4. Submucosa with Plexus submucosus, blood, and lymphatic vessels; 5. Mucosa.

The intraperitoneally located sigmoid and the retroperitoneal rectum are often involved in DE. Endometriotic nodules in the recto-vaginal septum result in adherence of the recto-sigmoid to the lower dorsal side of the uterus, cervix, and vagina. The large bowel wall consists of multiple layers, all of which can be invaded by endometriotic tissue (Fig.1). The sigmoid colon receives its blood supply from multiple branches of the inferior mesenteric artery. There exist anastomoses between the sigmoid arteries and the superior rectal (superior haemorrhoidal) artery, itself a branch of the inferior mesenteric artery, supplying the rectum and creating further anastomoses with the middle rectal (middle haemorrhoidal) artery, a branch of the inferior vesical artery which originates from the internal iliac artery. Distally, the rectum receives some blood supply from the inferior rectal (inferior haemorrhoidal) artery, which mainly supplies the anal canal originating from the internal pudendal artery, another branch of the internal iliac artery. Again, anastomoses exist between the middle and inferior rectal arteries.

**Figure 2:**
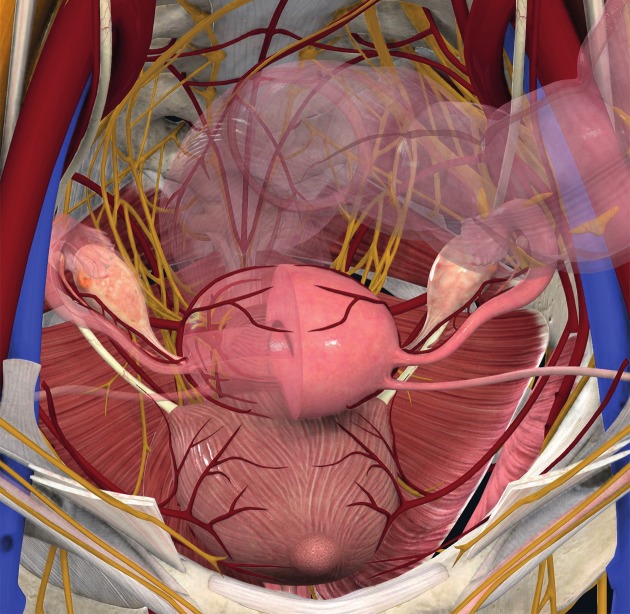
Anatomical landmarks in DE surgery (vessels, nerves, ureter, bladder, bowel) which may be involved and have to be respected carefully. DE: deep endometriosis. (Image courtesy of Complete Anatomy (reprinted with permissions).

Like any subperitoneal (deep) endometriosis, nodules in the vesico-uterine pouch (ventral cul-de- sac) can involve or invade surrounding structures, such as the underlying urinary bladder, eventually displacing the round ligaments medially. The bladder wall consists of mucosa, a submucosal layer (lamina propria) containing blood vessels and nerve fibres, a muscularis propria (detrusor muscle) consisting of an inner and outer longitudinal and a middle circular muscle layer, and either a serosal layer or adventitia. The ureters, after passing about 1 to 2 cm laterally to the uterine cervix and coursing ventral to the lateral border of the vagina, enter the bladder postero-laterally in an oblique angle. The urinary bladder receives its blood supply from the superior, middle and inferior vesical arteries (branches of the internal iliac artery) as well as the vaginal (branch of uterine artery) artery (see also Fig. 2).

#### Classification

**Figure 3:**
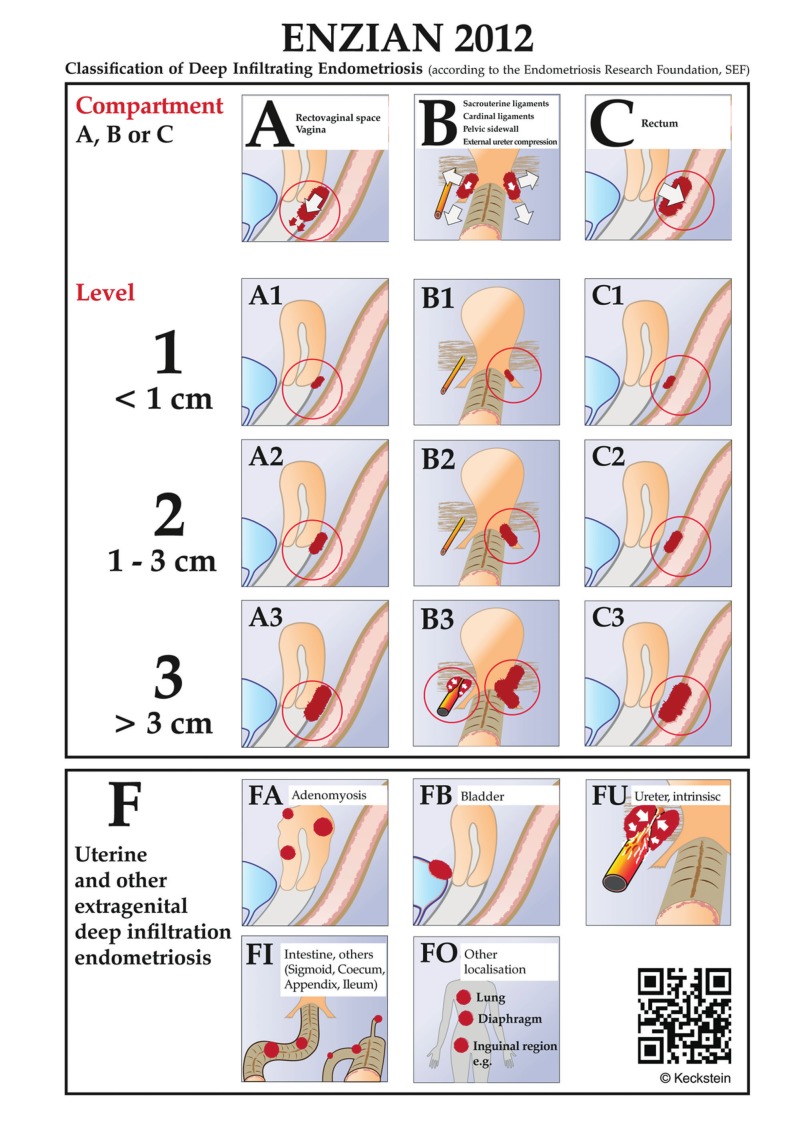
Revised ENZIAN-classification for DE. The system classifies the clinical findings of endometriosis according to their localisation (compartment) and size (<1 cm, 1-3cm, > 3cm). The ENZIAN classification focusses on the three dimensions (compartments) in the pelvis: A= craniocaudal axis or compartment (rectovaginal space, vagina), B= laterodorsal axis (uterosacral and cardinal ligaments), C= dorsal axis (rectosigmoid). Other localisations as uterus, bladder, ureter, other bowel involvement and extragenital localisations are respected as well and described with suffix F). The ENZIAN Classification is under revision (2019) again which is under publication.

The most commonly used classification system of endometriosis, the rASRM classification, does not provide sufficient information for DE; correlation with symptoms is poor and it does not predict surgical difficulty level or outcome (Johnson et al., 2017). Several systems classifying and documenting the extent of the DE have been developed, including the ENZIAN classification (Keckstein et al., 2003b; Tuttlies et al., 2005; Stiftung Endometriose Forschung (Foundatio for Endometriosis Research), 2011) (Fig. 3), the Visual Numeric Endometriosis Surgical Score (VNESS) system (Abdalla AL and S., 2015), and those proposed by Chapron et al and Adamyan (Adamyan, 1993; Chapron et al., 2003). The ENZIAN classification showed a significant correlation between the extent of the disease, difficulty and length of surgery, and symptoms (Haas et al., 2013a; Haas et al., 2013b; Haas et al., 2013c; Morgan-Ortiz et al., 2019; Montanari et al., 2019). The different scoring systems with their advantages and disadvantages are summarised in Table I.

**Table I:** — Classification systems for deep endometriosis.

Classification system	Scoring of compartments	Scoring of severity	Positive aspects	Negative aspects
ENZIAN Classification (Keckstein et al., 2003) (Tuttlies et al., 2005) (Stiftung Endometriose Forschung (Foundation for Endometriosis Research), 2011)	Retroperitoneal structures are divided into the following three compartments: - A: rectovaginal septum and vagina - B: Sacro uterine ligament to pelvic wall - C: rectum and sigmoid colonRetroperitoneal distant locations: - FA: adenomyosis - FB: involvement of the bladder - FU: intrinsic involvement of the ureter - FI: bowel disease cranial to the rectosigmoid junction - FO: other locations, such as abdominal wall endometriosis	Severity is rated in the same way for all compartments: - Grade 1: invasion <1 cm - Grade 2: invasion 1–3 cm - Grade 3: invasion >3 cm	Relatively good morphological description provided Correlation between symptoms and involved compartments Additional aspects can be calculated ( e.g. the anticipated operating time, risk of complication during and after surgery)	International acceptance of the classification is still low – mostly used in Europe
Chapron (Chapron et al., 2003)	Ventral DIE - A1: Bladder Dorsal DIE - P1: Uterosacral ligament - P2: Vagina - P3: Intestine (solely -with or without vaginal infiltration- or multiple intestinal location)	No scoring	Linked to an operative procedure	Reported in two publications by the authors, no further information Not widely used
Adamyan (Adamyan, 1993)	Adamyan Stage I: Endometriotic lesions are confined to the rectovaginal cellular tissue in the area of the vaginal vault. Adamyan Stage II: Endometriotic tissue invadesthe cervix and penetrates the vaginal wall, causing fibrosis and small cyst formation. Adamyan Stage III: Lesions spread into the sacro-uterine ligaments and the rectal serosa. Adamyan Stage IV: The rectal wall, recto-sigmoid zone, and rectouterine peritoneum are completely involved, and the rectouterine pouch is totally obliterated	No scoring		Not published in peer reviewed journal Not widely used
Visual Numeric Endo-metriosis Surgical Score (VNESS) (Abdalla AL and S., 2015)	Eight locations: Left adnexa – Left pelvic sidewall - Left utero-sacral area / ventral compartment (UVF) - Dorsal compartment (POD)/ Right uterosacral area – Right pelvic Sidewall - Right adnexa	For each location, a score between 0 and 4 representing the severity of endometriosis 0: No visible endometriosis 1: Superficial endometriosis 2: DIE with no attachment to viscera 3: DIE loosely adherent to viscera 4: DIE densely adherent to viscera OR invading muscularis OR kissing ovaries	Intuitive and easy to remember. Visually helpful	Not published in peer reviewed journal Not widely used

DIE; deep infiltrating endometriosis, UVF: Left uterosacral area / ventral compartment POD: Dorsal compartment

In addition to classifying endometriosis, the documentation of surgical findings, such as in Endometriosis Fertility Index (EFI), has been found to have a prognostic value in infertile women (Adamson and Pasta, 2010; Adamson, 2013).

The working group emphasises the importance of classification of DE, but also the limitations of the existing systems, specifically with regard to the scoring of the severity of disease. The ideal classification system for DE should define accurately the anatomical location, size of the lesions, and level of involvement of the adjacent organs. It should also be reproducible and should help the surgeon in the planning and execution of surgery.

The working group recommends documenting the following information:

- the location of DE lesions;
- uterosacral ligaments, including whether ureters are infiltrated;
- rectovaginal septum, including involvement of vaginal wall/mucosa;
- bowel, including involvement of muscularis layer;
- bladder, including involvement of muscularis and ureteral ostia;
- other sides in the pelvis;
- extrapelvic locations;
- involvement of the ovaries;
- the sizes of the lesions;
- the number of lesions;
- the degree of involvement of adjacent organs and structures.

The current paper is structured according to the different locations of DE, with further subdivision on extent (where relevant).

### Pre-operative assessment and preparation for surgery

#### 

Pre-operative assessment of patients with suspected DE aims to establish a diagnosis, evaluate extent of disease, and determine the optimal surgical approach. This includes a thorough medical history, clinical examination, and imaging. When assessing the medical history and symptoms, the focus should be directed on symptoms that could indicate the presence of DE lesions in specific organs/locations. These include, but are not limited to, cyclical haematuria and cyclical rectal bleeding (Chattot et al., 2019). It is very important to record co- morbidities and take them into consideration when deciding on surgery.

It is helpful to use a validated symptom questionnaire for data collection, for audit and comparison. Women’s symptoms, such as pain, should be assessed, for instance using a visual analogue scale and general quality of life questionnaires (e.g. the World Endometriosis Research Foundation (WERF) set up the Endometriosis Phenome and Biobanking Harmonisation Project (EPHect) Endometriosis Patient Questionnaire) (Vitonis et al., 2014; Bourdel et al., 2015; Vanhie et al., 2016). Other questionnaires can be used to assess specific aspects of endometriosis, including sexual function, urinary function, bowel function, recovery, and depression and anxiety. These symptoms should be assessed, taking into account uterine bleeding pattern and associated dysmenorrhea, as painful symptoms associated with irregular periods can be managed by starting and/or adapting a medical treatment.

Fertility plans and indications for surgery should be discussed before the surgical procedure, for instance using the EFI (Adamson et al., 2010). Choices of different treatment options (surgery, IVF) for DE and the selection of patients that would benefit from surgery are beyond the scope of this document. However, IVF could be proposed as the first step in patients who have been operated previously, who have a low ovarian reserve and/ or when there is male factor infertility (Dunselman et al., 2014). Oocyte freezing may also need to be discussed as an option in cases of coexistent ovarian endometrioma (Working group of ESGE-ESHRE and WES et al., 2017).

A surgical history is essential. Previous operation reports should be read in detail and any pictures/ videos reviewed carefully. The surgeon should know if the retroperitoneal space was opened and, if it was, its side should be noted. It is also essential to know whether ureterolysis and/or bowel dissection was performed. Extensive ureteral dissection always impairs ureteral vessels, so that even a very cautious and meticulous repeat ureterolysis may induce ureteral ischemia. Similarly, previous bowel dissection, and/or previous rectal shavings, implies that the bowel wall may be compromised by previous procedures, so the risk of bowel injury and/or postoperative fistula may be increased. As the risks of intra- and/or postoperative urinary or intestinal complications are considered much higher in women who have undergone previous extensive procedures, this should be taken in account during counselling the woman, decision-making, and organisation of the surgical team. History of previous ovarian cystectomy should be taken into account when planning and performing surgery on ovarian endometrioma in particular in women who may wish to conceive in the future (Working group of ESGE-ESHRE and WES et al., 2017).

#### Clinical examination

Clinical examination in women with suspected DE includes not only a physical examination of the pelvis but also the inspection and palpation of the abdomen. The examination may need to be extended beyond the pelvis, depending on the symptoms of the woman. Location and extent of disease can sometimes be determined by clinical examination (Ripps and Martin, 1992; Koninckx et al., 1996; Bazot et al., 2009). There should be special emphasis on the visualization of DE in the vagina by inspection of the dorsal fornix with a bivalved speculum.

Vaginal examination can facilitate the detection of infiltration or nodules of the vagina, uterosacral ligaments or pouch of Douglas. It could also contribute to the assessment of the extent of disease to the pelvic sidewall, which is important to evaluate the risk of trauma to the hypogastric plexus and/or the ureter. Rectovaginal digital examination may allow the detection of infiltration or mass involving the rectosigmoid or adnexal masses (Ripps and Martin, 1992; Koninckx et al., 1996; Eskenazi, et al., 2001; Condous et al., 2007). Rectal examination is highly recommended to assess the lateral and dorsal extension of the disease allowing detection of the patients who are at risk of hypogastric vessels injury and/or hypogastric plexus damage. It also allows the surgeon to evaluate the mobility of the nodule of the dorsal cul-de-sac and thus to predict how difficult the surgery may be.

#### Imaging and other investigations

##### 

**Figure 4:**
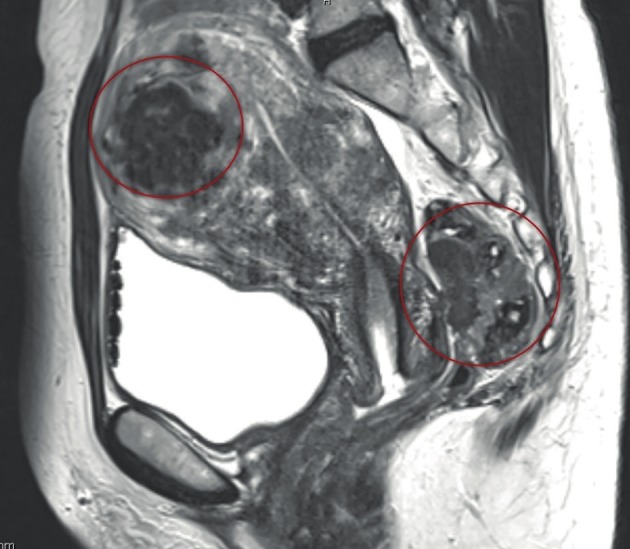
MRI picture of the pelvis. DE in the rectum with small, dense adhesions between the posterior wall of the cervix and ante- rior wall of the rectum (right circle) and adenomyosis (left circle).

The ESHRE Guideline on the Management of Endometriosis recommends assessing the ureter, bladder, and bowel involvement by additional imaging, if there is a suspicion based on history or physical examination of DE, in preparation for further management (Dunselman et al., 2014). Imaging for suspected involvement of bladder, bowel, and ureters may start with ultrasonography (US) (Guerriero et al., 2016). Other imaging techniques, such as MRI (Fig. 4) including neuro MRI, and computed tomography (CT) (only in selected cases as it is associated with unacceptably high radiation exposure) (Exacoustos et al., 2014; Guerriero et al., 2018) can also be used. Assessment of MRIs should be performed using high definition standards. Barium enema and sigmoidoscopy may give additional information about stenosis of the bowel. These investigations aim to determine the location, size, and number of DE lesions (nodules or plaques) as well as the level of infiltration (depth of invasion, length of infiltration, stenosis) into the organ/structure involved. Furthermore, the identification of lesions on/in the pelvic wall (i.e. sacral root) and other extragenital localisation (abdominal wall, inguinal canal, diaphragm, lung, etc) with specific imaging techniques is relevant, as it has an important impact on the surgical treatment and its planning. Kidney sonography is mandatory in every patient with DE potentially involving the ureters to prevent overlooking silent hydronephrosis.

##### Bowel

Where appropriate, involvement of bowel muscularis and the distance between the inferior border of the lowest bowel lesion and the anal verge should be evaluated as these would be expected to have an impact on the type of surgery that will be performed. Limitations of the accuracy of these investigations should be kept in mind.

**Figure 5:**
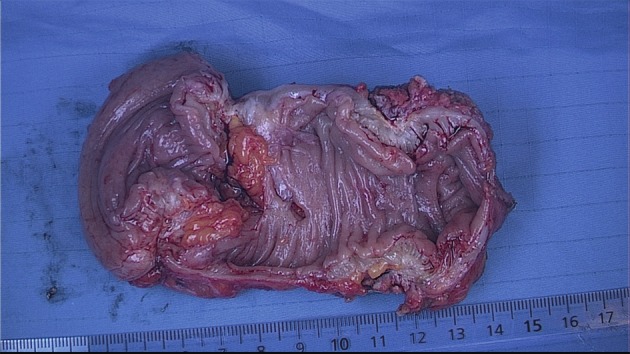
Deep endometriosis of the rectosigmoid. The extent of the white nodules (multifocal) which infiltrate the muscular layer are not visible by colonoscopy and also difficult to identify completely by laparoscopy.

Colonoscopy identifies stenosis or intraluminal lesions, which are rare, but unfortunately it does not give sufficient information about the presence, localisation and size of endometriosis in the bowel wall (Fig. 5).

**Figure 6:**
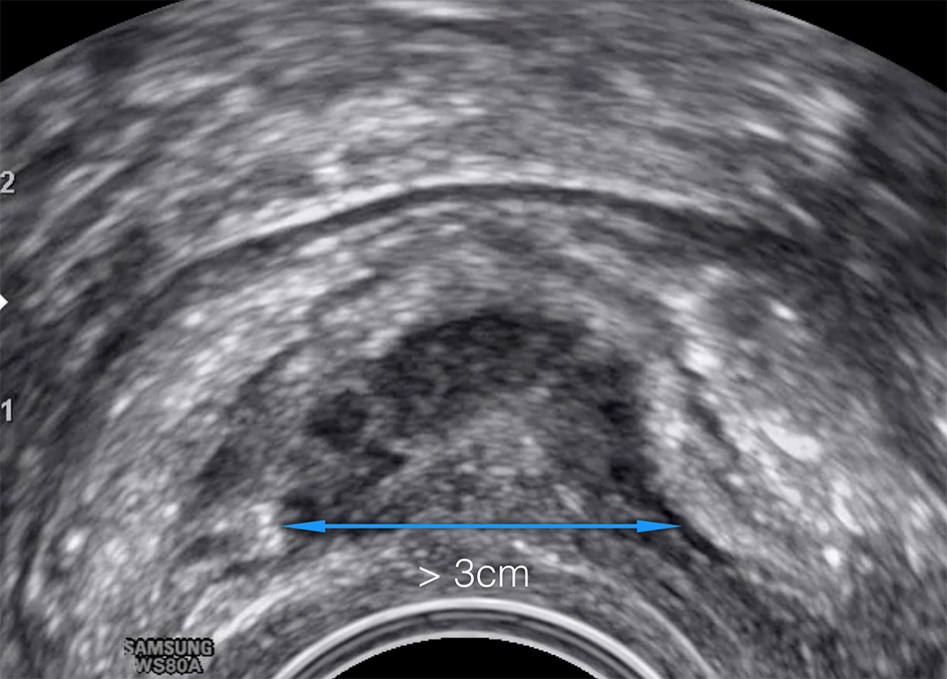
Transvaginal ultrasound of the rectosigmoid with signs of infiltration of the muscular layer. Length >3 cm Enzian C3. Hypodense ultrasound pattern (halfmoon shaped) represents the hyperplasia of the lamina muscularis with inclusion of epithelium, stroma and fibrosis.

US is the first-line imaging modality for the assessment of pelvic endometriosis. It has been demonstrated that most of the deep lesions in the lower colon can be identified with a high sensitivity and specificity (Hudelist et al., 2009; Hudelist et al., 2011; Exacoustos et al., 2017). It may have limitations with respect to field-of-view and operator dependence (Fig. 6).

Transrectal ultrasonography (TRUS) may be used for rectosigmoid involvement but could not be adequately assessed for other anatomical sites because of scant heterogeneous data.

MRI is usually performed as an additional examination in complex cases or prior to surgery and is highly accurate in the evaluation of endometriosis. Diagnostic accuracies were higher for transvaginal ultrasonography (TVUS or TVS) with bowel preparation (TVUS-BP), rectal water contrast (RWC-TVS) and for 3.0T MRI than for conventional methods, although the paucity of studies precluded statistical evaluation (Nisenblat et al., 2016). TVUS for DE is highly dependent on the experience of the operator and the quality of the US equipment. The additional use of vaginal and rectal contrast US gel can further enhance the image (Grammatico et al., 2017).

Multi-detector computed tomography enema (MDCT-e) is another technique which might have a high diagnostic performance for rectosigmoid and other bowel endometriosis (Ferrero et al., 2011).

Virtual colonoscopy may add new information to that provided by MRI (Mehedintu et al., 2018). Virtual colonoscopy is a single non-invasive short procedure. It provides information about lesions in the whole length of the colon (esp. sigmoid, ileum, caecum) (van der Wat et al., 2013). However, a dry bowel preparation is necessary, and the risk of irradiation has to be considered, as with the MDCT-e scan.

If rectal bleeding (haematochezia) is reported by the patient, colonoscopy is indicated for differential diagnosis of primary bowel disease.

##### Bladder

TVS is sufficient to diagnose bladder endometriosis in the majority of cases. Typically, lesions are located at the dorsal wall or the fundus of the bladder. They can extend into the vesico-cervical space and the ventral wall of the uterus. A partially filled bladder can optimize sonographic assessment, while a full bladder can make it more challenging (Guerriero et al., 2018). MRI is often not necessary to diagnose bladder endometriosis in addition to TVS but it can be helpful to identify the relation between the nodule and the ureteral ostia and if additional complex rectovaginal endometriosis is suspected (Carfagna et al., 2018; Aas-Eng et al., 2019).

Pre- or intra-operative cystoscopy is recommended for bladder endometriosis as it allows visualization of the blueish, submucosally protruding nodules. It is important to localize the lesion precisely in relation to the ureterovesical junction (UVJ) (Collinet et al., 2006; Fadhlaoui et al., 2015). Whilst the mucosa (urothelium) itself is rarely infiltrated, the nodule usually protrudes into the bladder cavity and may reach considerable size. Involvement of the outer layer of the detrusor muscle cannot be excluded by cystoscopy. A biopsy is only required for differential diagnosis when other diseases are suspected, such as urothelial carcinoma and/or interstitial cystitis. If surgery of bladder endometriosis is planned, the placement of ureteral stents can be advantageous.

Urodynamic evaluation to assess bladder function can be useful in case of bladder problems. It may have a place in distinguishing pre-existing bladder dysfunction from that developed postoperatively, as surgery for DE may induce de novo dysfunction, potentially caused by surgical nerve damage (de Lapasse et al., 2008). The use of a specific, validated questionnaires, such as International Prostate Symptom Score (I-PSS) (Barry et al., 1992) and Bristol Female Lower Urinary Tract Scale (BFLUTS) (Jackson et al., 1996), may improve the preoperative work-up.

##### Ureter and kidney

Assessment of the kidney is also necessary to rule out asymptomatic hydronephrosis (Lusuardi et al., 2012). Endometriosis of the ureter is very likely if hydronephrosis in women with endometriosis is present, so abdominal ultrasound is the gold standard in this situation. Hydronephrosis requires a functional work-up (side separate clearance) in order to assess the renal function. Imaging, including intravenous urography (IVU), high resolution TVUS, mercapto-acetyltriglycine (MAG3) renal scan (or radioisotope renography), MRI, and/ or contrast CT, is usually performed according to local protocols. Studies have shown the value of TV ultrasound scanning of ureters in patients with DE; in about 50% of the cases with ureteral involvement, obstruction can already be visualised by TVUS (cases lacking hydronephrosis - early stage obstruction) (Carfagna et al., 2018).

In case of hydronephrosis, the function of the kidney has to be checked before surgery.

##### Nerves

The involvement of nerves in DE is of great importance to the patient as well as to the surgeon (Possover et al., 2011; Chiantera et al., 2018).

Both the disease and radical removal of endometriosis may lead to the destruction of the nerve fibers with corresponding symptoms, which can be very debilitating for the patient.

Endometriosis close to the sympathetic and parasympathetic nerve fibers (hypogastric plexus and splanchnic nerves) can lead to a dysfunction of pelvic organs (e.g. dysfunction of the bladder as well as disturbance of vaginal lubrication and intestinal dysfunction) (Possover, 2014).

Involvement of somatic nerves, such as the sacral plexus and the sciatic nerve, leads to corresponding neurological symptoms or deficits.

In recent years, neuropelveology (Possover et al., 2015), a new field, has become established in endoscopy and gained great importance.

Laparoscopy provides an optimal surgical approach to the pelvic somatic nerves and allows micro- neurosurgery as a therapeutic approach. Both the exact pre-operative diagnosis in cases of suspected nerve involvement and the specialized surgical techniques to protect the nerves or even eliminate endometriosis close to the nerve are reserved for specialists who have undergone appropriate training in diagnosis and surgical treatment (Rabischong et al., 2018).

The current document draws particular attention to the importance of these procedures. However, these interventions require special training and detailed instructions on them would be the subject of another publication.

##### Extrapelvic lesions

The diagnostic approach for extra-pelvic endometriotic lesions includes physical examination (palpation), MRI, and US. MRI can help to visualise the extent and to plan the surgery. A complete diagnostic evaluation will minimise the risk of incomplete resection.

Abdominal wall endometriosis including scars (secondary to Caesarean sections or hysterectomy using open route), the umbilicus and the inguinal region are thoroughly explored using either low depth US or MRI examinations. The size and depth of nodules, and the involvement of muscles or aponeurosis, should be checked before the surgery in order to maximise the chances of a complete excision. Large defects after excision have to be closed with the help of a mesh.

MRI (especially high definition) may reveal endometriosis of the diaphragm; usually when the lesions are larger than 5mm, or when they presented recent bleeding (T1 frontal, axial, and sagittal views). However, small lesions may be overlooked during the pre-operative assessment, and may be only intra-operatively revealed. When laparoscopic examination of the diaphragm is carried out using a trans-umbilical endoscope, the surgeon should be aware that only the ventral part of the diaphragm can be explored. However, lesions located behind the liver in the hepato-phrenic cul-de-sac are routinely associated with visible satellite lesions spread on the ventral part of the diaphragm (Ceccaroni et al., 2012).

#### Informed consent

Informed consent should be relevant to the patient and must cover the extent of the surgery that may be performed and its potential complications. Pros and cons of alternatives to the proposed treatment must be presented (Bolton, 2015). Short- and long-term side effects of surgery should be explained. The use of current patient information leaflets or evidence- based online resources, with references/links to best practice guidelines, should be considered to provide a sufficient source of information that the woman can review in her own time. This may also be helpful as evidence of appropriate pre-operative counselling.

#### Multidisciplinary surgical team

The surgical team should be organised according to the needs of the planned or anticipated procedure(s). A bowel surgeon, a urologist, a thoracic surgeon, and even a plastic surgeon may need to be involved. The gynaecologist should lead the team as his/her understanding of endometriosis and the woman’s symptoms and needs is crucial in planning the surgery. The gynaecologist advocates for the patient and sets up a unique care plan, together with the team and patient, to improve the woman’s pain, fertility, and quality of life. Such a care plan should consider input from other disciplines related to the technical aspect of procedures. A multidisciplinary team meeting before the surgery may be helpful. The team should be informed well in advance in order to plan the procedure and to organise their time adequately.

If an ileostomy or a colostomy is planned, this should be discussed extensively with the woman and the site of the stoma may be decided and eventually drawn on the skin before the procedure.

#### Pre-operative strategies for a safe and complete excision of the lesion

##### Bowel preparation

Different types of bowel lumen cleaning can be helpful in cases of lesions in the dorsal compartment for the following reasons:

- an empty bowel gives more space in the pelvis during the dissection;
- the use of rectal probes or manipulators in a clean bowel will cause less contamination with faeces on the perineum, especially when the vagina has to be opened;
- in the case of opening the bowel, it will minimise faecal soiling in the abdomen.

According to the literature data on colorectal surgery, mechanical bowel preparation and enemas are widely used (Guenaga et al., 2011; Oliveira et al., 2016), though specific trials on the usefulness of bowel preparation in endometriosis surgery (where the vaginal fornix dorsal is often opened) are not available. Whereas none of these techniques have a proven benefit on the reduction of postoperative complications (Guenaga et al., 2011), in cases where a low anastomosis is expected mechanical bowel preparation is better than an enema (Platell et al., 2006). The use of intraluminal antibiotic decontamination of the bowel to reduce clinically relevant anastomotic leakage can be considered (McDermott FD et al., 2016).

##### Ureteral stenting

Pre-operative placement of ureteral stents is suggested when: surgery of large bladder endometriosis is planned; ureteral endometriosis is suspected pre- operatively; hydronephrosis is present; or there is a history of previous ureteral surgery.

##### Uterine manipulator and rectal probe

**Figure 7:**
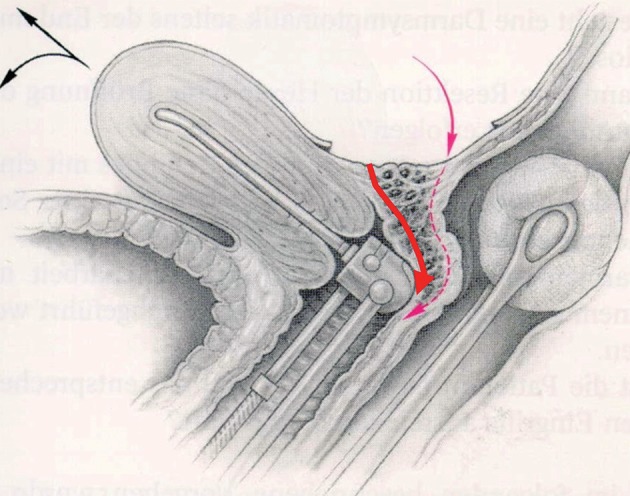
Deep endometriosis in the vagina, rectovaginal septum and the anterior wall of the rectum (Enzian A,C compartment). Two different ways to excise the nodule. A manipulator and a sponge or rectal probe is in situ for a better presentation of the nodule during the excision procedure. (Reprinted with permissions from Keckstein and Hucke, 2000).

**Figure 8:**
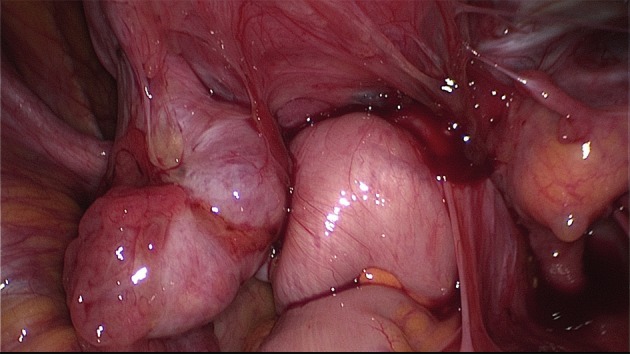
Frozen pelvis with invisible deep endometriosis (bi-lateral ovary, left cardinal ligament, ureter left, anterior wall of the rectum).

The use of a uterine manipulator achieves maximum mobility of the uterus, thereby improving visualization and facilitating dissection. A rectal probe could also be helpful in moving the bowel, although it could be hindered by stenosis due to a deep nodule or severe bowel adhesions. Tactile feedback between the dissecting instruments and the rectal and vaginal probes, handled by an assistant, helps to identify the correct planes of cleavage. Bowel and adnexal suspension can improve visualization and access to the pouch of Douglas (Einarsson and Wattiez, 2016) (Figs 7 and 8).

#### Strategy of the surgical intervention

Each surgeon needs a strategy for the operation, which is influenced by many factors including the size, activity and localization of endometriosis as well as the age and expectations of the patient, and the results of previous interventions.

Advanced endometriosis in a young patient with a desire to have children may be operated differently than in a patient over 40 years of age with pain as the main symptom.

The surgeon is often confronted with the conflict between complete removal of endometriosis and the need for preservation of organs affected by the disease.

Another challenge is tackling multi-organ involvement, which requires a complex intervention possibly in a multidisciplinary setting

An important limitation is the risk of postoperative complications. For this reason, occasionally a limited radicality or surgery in several steps may be chosen, for example simultaneous segmental resection of intestinal endometriosis and ureteral re-implantation in hydronephrosis may be avoided.

Decisions for the strategy before and during surgery depend in particular on the situs and surgeon’s experience, and they have to be made on a case-by-case basis.

#### Open versus endoscopic surgery (or robotic assisted)

Endoscopic access has become standard for the treatment of endometriosis, including DE. It is obvious that the endoscopic procedures are advantageous due to better views and access to lesions in the depth of the pelvis, as well as lower postoperative morbidity. Minimal trauma to the abdominal wall and the healthy peritoneum, lack of dehydration and the use of microsurgical techniques improve the outcome, especially in patients with infertility. Furthermore, there are anatomical sites or endometriosis findings that can exclusively be reached/treated only by an endoscopic procedure (e.g. neuropelveology).

Endoscopic operations require special instruments and equipment as well as a high level of training and experience of the surgeon. The access route should be chosen according to the clinical findings and the existing options.

However, a laparotomy (sometimes with midline incision) may occasionally be more effective than several inadequate laparoscopies. The advantage of a laparotomy for treatment of severe endometriosis to identify and completely eliminate it lies in the ability of having a better tactile feedback. In this situation, microsurgical operation techniques should still be used.

Robot-assisted surgery has gained importance in the treatment of endometriosis over the last 10 years. Special features of the instruments may facilitate difficult steps of the procedures and some of the benefits of laparotomy are thus incorporated into endoscopic surgery. Several studies have shown that the results of robot-assisted surgery in DE are equivalent to those of conventional laparoscopy, but not superior.

In the current document, particular attention is given to conventional endoscopic procedures.

### Initial steps of DE surgery - patient positioning in view of anticipated long duration of surgery

Prevent pressure sores and compartment syndrome by using:

- anti-embolism stockings for thromboprophylaxis, and additional prophylaxis with postoperative low molecular weight heparin is usually recommended after this type of pelvic surgery (follow local guidelines);
- body warmer to maintain the core temperature;
- boots, lithotomy with soft stirrups, legs flat, intermittent pneumatic compression devices.

The woman is placed in the modified dorsolithotomy position and her legs are placed in surgical stirrups carefully avoiding trauma to the leg nerves. As the surgery is often long, particularly in women with previous multiple surgeries and/ or in obese or moderately overweight patients, mobilization and/or massage of the legs can be performed every 2-4 h or between different surgical phases. Application of intermittent pneumatic compression devices or sequential compression devices has been proposed to limit the risks of lower limb compartment syndrome (Tomassetti et al., 2009; Gould et al., 2012). Arms should be positioned carefully to avoid shoulder restraints or pressure, especially in steep Trendelenburg position during surgery.

- Examination under anaesthesia is generally recommended for DE. An additional rectovaginal exam under laparoscopic vision may be beneficial particularly when rectovaginal nodules are not obviously visible.
- Antibiotics can be used according to local guidelines.
- Systematic laparoscopic inspection and documentation is recommended. After insertion of the laparoscope, the upper abdomen including diaphragm and appendix/caecum should be inspected, preferably prior to placing the patient in (adequate) Trendelenburg.
- The placement of secondary trocars for the various instruments should be individualized according to the anatomical situation and surgical needs.

The basic principles to identify and treat deep endometriotic lesions are stated in Table II.

**Table II:** — Principles for identifying and treating deep endometriotic lesions.

☐ Identify all important anatomical structures (ureters, colon, small bowel, major vessels, adnexae, uterus, bladder, nerves).	
☐ Identify the lesions.	
☐ Signs of deep endometriosis include:	
	☐ fibrosis, with or without characteristic dark spots
	☐ (dense) adhesions
	☐ distortion of anatomical structures, infiltrations
	☐ reduced tissue elasticity
	☐ haemorrhagic cystic structures
☐ Perform easy steps first as this will facilitate difficult ones.	
☐ Divide adhesions and restore pelvic anatomy in addition to complete excision of endometriosis.	
☐ Free and isolate the lesions.	
☐ Start the dissection in areas free of disease.	
☐ Optimise exposure by using manipulators, ovariopexy, and additional ports, if necessary.	
☐ Aim for complete excision whenever reasonable and possible. *	

* When deciding that a part of the disease may be left behind, the surgeon should remember that if an extensive dissection has been performed to access this part of the disease, reoperation will be extremely difficult and sometimes almost impossible. If excision is considered to be too risky, it is likely to be even more difficult and dangerous if a reoperation is needed due to recurrent pain or other severe symptoms (such as stenosis of the bowel or ureter). Ideally, surgeons would be prepared to manage all aspects of the disease.

### DE of the uterosacral ligaments and rectovaginal septum with or without involvement of the rectum

#### 

**Figure 9:**
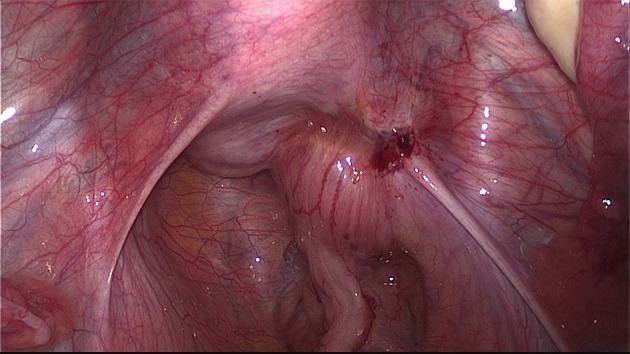
DE involving the uterosacral ligament, vagina and the rectum. The extent of the disease is not visible during diagnostic laparoscopy. Spaces have to be opened in order to get access to the entire lesion.

**Figure 10:**
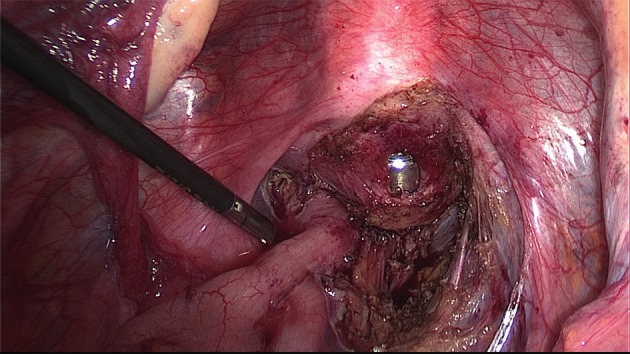
Dissection of the posterior compartment. Right uterosacral ligament has already been resected, the vagina is partially open with the manipulator in place.

The extent of the surgical procedure is determined by the size of the lesions, their location, their number (single or multifocal), and the degree of infiltration (Figs 9 and 10).

#### Definitions of bowel infiltration and surgical procedures

The pre-operative clinical diagnostic workup including clinical examination (vaginal and rectal), TVUS, and an optional MRI is necessary in order to identify the bowel wall infiltration pre-operatively. A negative colonoscopy does not exclude the intramural presence of DE.

In case of muscularis infiltration by the nodule, the distance of the inferior border of the most distal bowel lesion to the anal verge should be evaluated, as this may impact on the type of surgery performed.

If the lesions are only located in or on the serosa without infiltration of the muscularis layer, it can be treated with superficial resection (serosal shaving) (Koninckx et al., 2012; Vanhie et al., 2016).

If deep (infiltrating) lesions involve the muscularis layer, sometimes the submucosa and even the mucosa, partial or full thickness removal by shaving, discoid, or segmental bowel resection is necessary (Koninckx et al., 2012; Donnez and Roman, 2017) (Figs 5 and 7).

#### First steps of surgery

##### Uterine, vaginal, and rectal set up

A uterine manipulator is used to improve exposure of the cul-de-sac, particularly in the presence of an enlarged uterus due to fibroids and/or severe adenomyosis, which makes the procedure more difficult. In some cases, a sponge can be placed in the dorsal fornix of the vagina. A rectal probe should also be available to mobilize the rectum in order to determine its position and attachment to the vaginal wall and to evaluate the elasticity of the tissue and degree of stenosis, taking care to avoid inadvertent rectal laceration. During the surgery, these three manipulators can be mobilized individually in order to clearly identify the limit between the vagina, the rectal wall, and other pelvic structures (Figs 7, 9 and 10).

##### Preparing the operating field

###### 

Prior to starting the operation, a vaginal exam using a bivalved speculum and digital (vaginal and rectal) examination for the evaluation of the dorsal fornix (mucosa protrusion/retraction or invasion) and the extension to the pelvic sidewalls is recommended.

The following steps may facilitate the surgical procedure: ovariolysis and ovariopexy, sigmoid mobilization, ureterolysis, and the identification of ligaments and rectosigmoid colon.

##### Ovariolysis

The mobilization of fixed ovaries on the pelvic sidewall improves the view of the operative field, particularly the lower structures in the cul-de-sac, which simplifies the identification of the ureters. If present, endometrioma should be drained and managed according to previous recommendations (Working group of ESGE-ESHRE and WES, et al., 2017). This may be done immediately after the drainage of the cyst or after having removed all other deep lesions in the pelvis. Endometriosis in the ovarian fossae should also be removed.

##### Temporary ovariopexy

Suspension of the ovaries with sutures (curved or straight needle through the abdominal wall) or special devices can maximize access to the pelvic structures and especially the pararectal spaces and the pelvic sidewalls.

##### Mobilisation of the sigmoid: (starting from the ‘white line of Toldt’)

Mobilize the sigmoid colon off its attachments to the abdominal wall and pelvic sidewall to expose the left adnexa and underlying structures such as the left pararectal space and ovarian fossa.

##### Ureterolysis

It is advisable to identify the position of the ureter on the pelvic rim or upper pelvic sidewall at the beginning of the procedure. The ureter should be followed down to the cardinal ligament and the crossing with the uterine vessels. When covered with endometriotic lesions, dissection of the ureter will be necessary to prevent inadvertent damage. The procedure can be simplified with the previously performed ovariopexy.

##### Bowel adhesiolysis

The approach to the dorsal compartment is often hindered by dense adhesions between the rectum, uterosacral ligaments, and the dorsal side of the uterus (Fig. 8). Even with the use of manipulators it may be very difficult to identify the exact planes of cleavage for the dissection procedure.

As the presence of dense adhesion between the rectum and other structures may obscure the DE, complete dissection of these adhesions is mandatory. Opening of the pararectal fossa–starting in healthy tissue– will facilitate the excision of the nodule.

Adhesiolysis is performed with cold scissors, blunt dissection, or thermal instruments with minimal collateral thermal spread.

Aquadissection can be considered in special situations: injection of Ringer’s Lactate solution with or without diluted vasopressin may help to identify the planes of cleavage and to separate vital structures, such as the ureter or bowel, and may reduce bleeding (oozing). The liquid can be injected with a spinal needle inserted individually and directly through the abdominal wall, or with other instruments such as suction/irrigation cannulas.

### Second step of surgery for DE involving the rectovaginal space

#### DE involving the muscularis layer of the rectum with no vaginal infiltration

In such cases, shaving consists of the separation of the DE nodule from the ventral part of the rectum.

The aim of the procedure is to mobilize the ventral rectal wall from the nodule until the rectum is free (first lateral then central dissection until finally the distal border of the nodule is released) by means of mechanical dissection (cold scissors) or low-thermic energy sources (e.g. CO2-laser, plasma).

Pararectal spaces are opened longitudinally, medially from the uterosacral ligaments, and as close to the lateral side of the bowel as possible in order to avoid injury to the hypogastric and splanchnic nerves. Dissection is continued until the opening of the healthy rectovaginal space. The use of a uterine manipulator and/or a vaginal probe helps to identify the important structures, such as the vagina, the cervix and the rectovaginal septum, by moving it in various directions.

Once the lateral sides of the rectum are freed, rectal shaving is performed on the ventral wall of the rectum to remove the endometriotic lesion completely. Thus, the nodule is dissected away from the rectal wall in an upward direction, resulting in the rectum falling back dorsodorsally. The nodule is then dissected off the dorsal cervix, the uterosacral ligaments, and the vagina without opening the latter in the absence of infiltration.

An alternative surgical technique named “the reverse technique” can also be applied, in which the DE lesion is separated first from the cervix and the vagina and only in a second stage from the rectum (Kondo et al., 2011, Bourdel et al., 2018). The removal of the nodule from the mobilized rectum can be facilitated with a probe or sponge inserted transanally into the lumen (Fig. 7).

The advantage of the reverse technique could be that any opening of the intestinal lumen takes place very late and hence the contamination time of the surgical field is shorter.

After the complete excision of the nodule from the wall of the bowel, leaving soft tissue behind, it is always required to check the integrity of the bowel wall. This can be done by gently stretching the bowel over the rectal manipulator to identify thinned areas. Another method to detect leakage is to administer air into the rectal lumen while the pelvis is filled with water or to fill the bowel with diluted methylene blue. In the near future, the use of indocyanine green (often used by general surgeons when assessing bowel anastomosis) will have to be considered, adding an evaluation of the vascularisation of the bowel wall to the identification of a leakage (Bar-Shavit et al., 2018; De Neef et al., 2018; Seracchioli et al., 2018; Shen et al., 2018).

In the case that a muscularis/partial thickness defect is identified, this can be sutured in one layer by using absorbable stitches starting in healthy margins. In the case of a full thickness defect (opening of the mucosa), a two-layer technique or a conversion to disc excision using a transanal stapler, which provides a tight stapled line involving healthy rectal wall, is suggested.

The disadvantage of the shaving technique in the infiltration of the muscularis is that it may result in incomplete excision, which should be considered in the pre-operative counselling of the woman.

#### DE involving the muscularis layer of the bowel and the vagina

In the case of vaginal infiltration by the DE nodule, the first step of the procedure is similar to that previously described (see DE involving the muscularis layer of the rectum with no vaginal infiltration). The aim is to resect the vaginal fornix adjacent to the uterine torus and to the ventral root of the uterosacral ligaments (Fig. 7). This may be aided by the use of a manipulator in the uterus and/ or vagina (sponge, manipulator). Where possible, preserve a rim of healthy vaginal mucosa attached to the cervix in order to facilitate the vaginal closure. The DE nodule can be extracted through the vaginal opening. Before starting to suture the defect, the completeness of the excision should be checked by a vaginal examination.

The excision of the vaginal nodule may also be done via the vaginal route as a first surgical step under laparoscopic guidance (Possover et al., 2000). The suturing of the vaginal defect can be performed laparoscopically or vaginally by using running or interrupted sutures (e.g. absorbable braided suture). When suturing laparoscopically, the pneumoperitoneum can be maintained by inserting a roll of swabs (potentially covered by a glove) into the vagina.

### Second step of surgery for DE involving the uterosacral ligaments, cardinal ligaments, and pelvic sidewall

#### No infiltration of the ureters

##### Uterosacral ligaments

The operative treatment of lesions involving the uterosacral ligaments depends on the extent of the disease and the reactive alteration of the tissue. These lesions should be removed completely with scissors or low thermal instruments, since coagulation alone may not be deep enough and therefore incomplete. Prior to the excision, it is advisable to identify the landmarks on the pelvic wall, which are the ureter, bowel, nerves, and pelvic vessels, and to dissect these structures carefully if involved in the disease process. The ureter usually runs closely to the lateral side of the uterosacral ligament and can be densely adhered to it. Medially, mobilization of the rectosigmoid will provide more space and preparation is carried out in the direction from dorsal to ventral. Once the uterosacral ligament is exposed dorsally, it can then be fully mobilized and excised towards the uterus. An important aspect here is the proximity to the hypogastric nerves, which should be avoided if not involved, or largely spared by a meticulous dissection technique. In the case that hypogastric nerve fibres are involved, a complete excision would include the resection of these as well. In the case of bilateral involvement of the hypogastric nerve, a more conservative approach should be considered to preserve bladder and sexual function.

To protect the hypogastric nerves, it is advisable to prepare them cranially in order to protect them in the area of the uterosacral ligaments.

##### Cardinal ligaments

If the parametrium is involved, the uterine vessels and the splanchnic nerve fibres should be conserved accordingly. The exposure of these structures can be achieved by dissection of anatomical spaces such as the Latzko space, the Okabayashi space, and the Yabuki (Fourth) space.

The mobilization of the lesions usually starts cranially and laterally in the parametrium, directed towards the medial side. In the case of extensive involvement, it may occasionally be necessary to sacrifice the uterine vessels, although the latter should be avoided in women desiring pregnancy after surgery (especially bilateral); this may mean that parametrial excision is incomplete.

The depth and the lateral extent of the preparation increases the risk of harming the parasympathetic nerves significantly.

It may also be necessary to remove the insertion of the cardinal ligaments at the outer part of the torus to complete the surgery. This radical approach should be avoided in the instance of a bilateral involvement of the parametria.

If the cardinal ligament involvement reaches the pelvic sidewall, the dissection procedure should be started in healthy tissue close to the internal iliac vessels, sacral root, and (para)sympathetic nerves. In such cases, neuropelveologic knowledge and advanced experience in radical surgery in this area is mandatory.

#### Infiltration of the ureters

If the lateral parametrium/cardinal ligament is involved, one strategy could be to remove the nodule in two parts (transecting it): first the rectovaginal part, and secondly the parametrial part since at that point the anatomy will have become clearer, especially with regards to the view of the nerves and vessels.

The obstruction of the ureter with consecutive hydronephrosis makes an intervention mandatory.

The ureter has to be freed completely by excision of pathologic structures within the ligaments and connective structures lateral and dorsal to the uterus. It is important to use either cold scissors or low thermal energy sources (e.g. CO2-laser, plasma) when dissecting the ureter in order to minimize the risk of damage to its microvasculature.

Though, in most cases, the ureter is narrower due to external pressure from fibrotic tissue, in 2-5% of cases a true infiltration of the intrinsic ureter wall is present and segmental resection with an end-to-end anastomosis or re-implantation into the bladder may become necessary (see also Urinary tract endometriosis).

### Second step of surgery for DE involving the bowel

#### 

**Figure 11:**
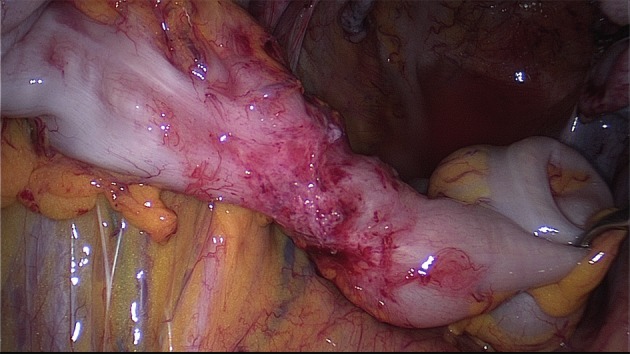
Symptomatic DE of the sigmoid colon with stenosis. Segmental resection is necessary.

In the case of bowel infiltration, several procedures could be considered according to the consent of the woman (Keckstein et al., 2003a; Tuttlies et al., 2005; Koninckx, et al., 2012; Abrao et al., 2015; Roman et al., 2016a; Abrao et al., 2017; Donnez and Roman, 2017) (Fig. 11).

#### Discoid excision

If the rectal wall is still infiltrated by implants of DE after shaving, it will appear hollow, rigid, and thickened when palpated with a laparoscopic probe and/or a rectal probe.

In these circumstances, to achieve a macroscopically complete excision, a full-thickness discoid excision of the shaved area may be performed, followed by suturing the defect in one or two layers.

Before performing a discoid excision, the extent of the bowel circumference involvement has to be evaluated. Indeed, there is a correlation between the depth of bowel infiltration and the circumference of the bowel affected by the disease.

**Figure 12:**
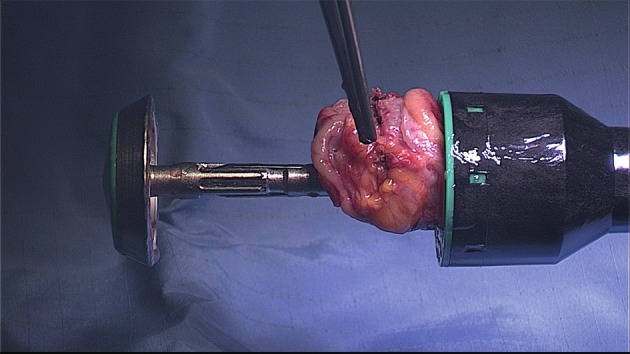
Disc resection of the anterior rectal wall with the circular stapler. Specimen in the open stapling device.

**Figure 13:**
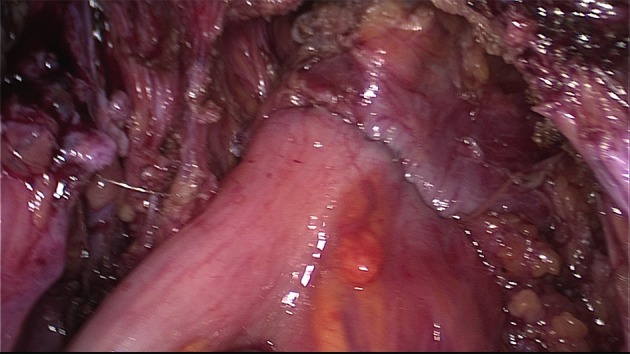
Final view of the rectum with the lining of the stapler in the anterior wall.

**Figure 14:**
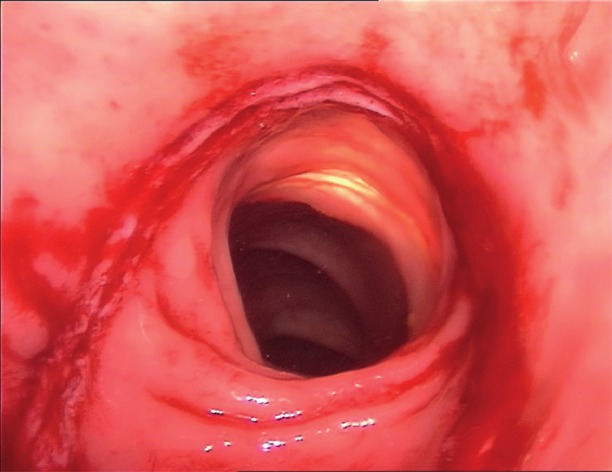
Rectoscopy with the view of the stapler lining in the anterior rectal wall.

Instead of suturing the defect in one or two layers, discoid excision can also be performed by using transanal staplers (either semi-circular or end-to-end circular staplers) (Figs 12, 13 and 14).

The semi-circular stapler allows large discoid excision (5-6 cm diameter on average) when the shaved area is located between 8 and 10 cm above the anus. The end-to-end circular stapler can also be used to remove discoids up to 3 cm in diameter located in the upper rectum and rectosigmoid junction. Preliminary rectal shaving reduces the thickness of the rectal patches, facilitating the excision (Roman, 2013).The rectal wall is pushed with a probe or pulled with threads into the head of the open stapler, which is then closed. In case that the resection of the lesion was incomplete (owing to the size of the lesion or incorrect placement of the stapler), a second full thickness resection is possible (to remove the residual lesion including also the first stapler line) (Kondo, et al., 2015, Trippia, et al., 2016, Braunschmid, et al., 2017).

#### Colorectal resection

**Figure 15:**
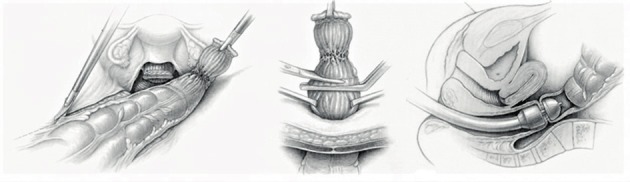
SDE of the rectosigmoid colon. Segmental resection with linear stapler, resection of the segment outside of the abdominal cavity is followed by the anastomosis with circular stapler (Reprinted with permissions from Keckstein and Hucke, 2000).

Dissection is carried out through the rectovaginal septum and follows the steps described above (Keckstein and Wiesinger, 2005; Abrao et al., 2015; Bouquet de Joliniere et al., 2019) (Fig. 15).

Mobilization of the rectum is carried out at least 20 mm below the rectal nodule.

The proximal dissection line is close above the lesion. Mobilisation and dissection of the bowel from the mesorectum and mesocolon is performed in contact with the dorsal wall of the rectosigmoid, which offers the possibility to preserve the mesorectum and mesosigma. This technique is used especially for short segments and if no other extraperitoneal structures are involved. When preparing the intestine this way, care must be taken not to damage the microvasculature of the intestinal wall due to thermal injury (Hudelist et al., 2018).

In the case of multiple rectosigmoid lesions, these may be removed en bloc using a long segmental resection, or rectal discoid excision could be associated with short segmental resection of the sigmoid colon in order to spare healthy bowel located between the nodules: the latter technique requires a very careful check of the blood supply of the bowel.

The stapler is entered into the peritoneal cavity through one of the inferior trocars and the rectum is then distally sectioned. A mini-laparotomy of 4 cm is carried out in a transverse suprapubic fashion (Pfannenstiel incision) or at the place of the inferior left or right laparoscopic port site.

After the mobilized intestinal segment is pulled in front of the abdominal wall, the resection of the involved part of the bowel takes place, which is performed proximally close to the macroscopic nodule.

The extraction of the bowel through the opened vaginal vault during hysterectomy, or an already opened vaginal fornix, is another option but this requires a long segment of the bowel to be mobilized. With this technique it should be noted that the farther cranial intestinal sections are difficult to access by palpation, even with resection techniques that are performed laparoscopically without the aid of a mini-laparotomy. Therefore, there may be a risk of missing small or additional “difficult-to-identify” nodules and, consequently, leaving them behind after surgery.

The transrectal extraction of the proximal segment has been described by individual authors. However, this requires that the intestinal lumen is open and thus also a higher risk of contamination for the abdomen exists during this more complex procedure.

The anvil of the circular stapler is introduced into the stump of the colon and fixed. After the stump has been brought back into the pelvis the anastomosis is performed by using end-to-end or end-to-side anastomosis with circular transanal staplers.

Care should be taken to avoid tension on the anastomosis. Especially in case several staple magazines have been used and thus staple lines may overlap, the risk of a leakage increases. Sufficient blood supply of the bowel wall needs to be ensured (Braunschmid et al., 2017).

If the sigmoid alone is affected, and there is a dolichosigmoid (i.e. elongated sigmoid), there is also the possibility to mobilize the intestine and to exteriorise the affected segment through a mini laparotomy for inspection and to perform segmental resection conventionally with a hand-sewn anastomosis (Fig. 11).

With simultaneous resection of the dorsal vaginal fornix and intestinal anastomosis the application of an omentum flap may be considered, although the effect on postoperative infertility remains unclear.

At the end of the procedure, the rectal air test, or the injection of dye (methylene blue) into the rectum may be used to control the quality of colorectal suture and the absence of leakage.

A diverting stoma may temporarily be created in women with concomitant rectal and vaginal sutures or with very deep anastomosis. The use of a surgical drain close to the anastomosis is recommended.

Though a stoma does not always ensure primary healing, it reduces the risk of a fistula formation with faecal peritonitis (Mabrouk et al., 2015). An early closure of a stoma (4 weeks after surgery) is possible in an uneventful postoperative process. However, performing a stoma is related to specific complications and the need for additional procedures to solve them (8.6% Clavien Dindo III complications in (Bonin et al., 2019)), thus the patients should be informed about these pre-operatively.

#### Low anterior rectal resection

Before performing a colorectal resection, the distance of the inferior border of the lowest bowel lesion to the anal verge must be discussed pre- operatively. Indeed, the surgical treatment of low rectal lesions (defined as 5–8 cm from the anal verge) is associated with a higher risk of postoperative anastomotic leaks (Ruffo et al., 2010) and transient neurogenic bladder dysfunction (Dousset et al., 2010). The patients should also be informed about the risk of low anterior rectal resection syndrome (LARS) as this can have significant negative consequences for gastrointestinal functioning and overall postoperative quality of life.

A newer technique combining a laparoscopic and transanal approach can be applied to remove the full thickness of the infiltrating endometrial nodules of the lower and middle rectum (Wolthuis et al., 2011; Bridoux et al., 2012). This technique may reduce the risk of rectal stenosis and denervation (Pronio et al., 2007; Goncalves et al., 2010; Bridoux et al., 2012; Roman and Tuech, 2014).

#### Other intestinal interventions

Nodules involving the ileocecal valve, the appendix, and the small intestine are often found next to rectosigmoid lesions. Due to the multifocal and multicentric occurrence of endometriosis, the entire intestine (appendix, small bowel, caecum, and ileocecal valve) should always be inspected.

The extent of this type of endometriosis is difficult to visualize completely by laparoscopy. If suspected, mobilizing this entire intestinal section will allow for it to be exteriorised through a mini-laparotomy in the right lower abdomen or by extending the umbilical incision. The resection is done conventionally (hand-sewn) or with stapler technology.

### Urinary tract endometriosis

#### Bladder endometriosis

##### 

DE of the bladder is often associated with an involvement of the detrusor muscle and -rarely- infiltration of the mucosa (urothelium). Involvement of the lower urinary tract is found in 0.2-2.5% of all DE cases, although rates up to 52.6% have been reported (Knabben et al., 2015; Acker et al., 2003; Le Tohic et al., 2009; Fadhlaoui et al., 2015). Of these, bladder and ureteral lesions, respectively, occur in 25-85% and 15-75% of the cases, while renal and urethral involvement is even rarer (5%) (Acker et al., 2003; Carmignani et al., 2009; Fadhlaoui et al., 2015; Badri, et al., 2018).

Before starting surgery for bladder endometriosis, information on the location of the lesion is important. The presence of hydronephrosis provides additional information as to the potential involvement of the ureters and the pelvic sidewall, respectively.

More details on imaging techniques and pre- operative procedures are available in the section on pre-operative management. It must be mentioned that, in a majority of cases, the pre-operative assessment does not allow for accurate prediction of the feasibility of the ureterolysis and the need for ureteral resection. Severe hydronephrosis with kidney atrophy may ultimately be due to ureteral extrinsic endometriosis, which can be treated by ureterolysis.

##### Surgical management

**Figure 16:**
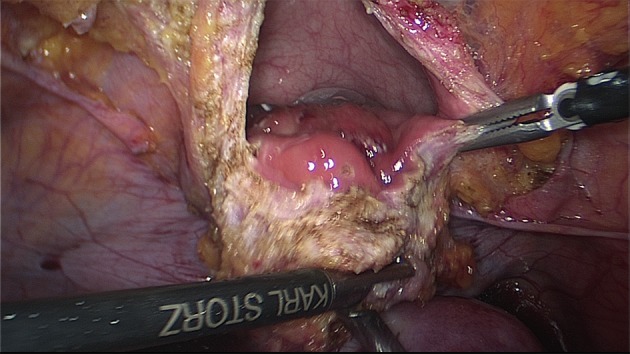
Deep endometriosis of the bladder.

**Figure 17:**
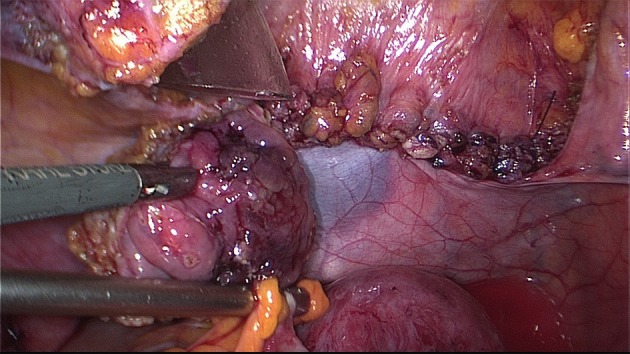
DE of the bladder. The cystotomy was closed transversally with 3-0 resorbable running suture. The nodule (3 cm diameter) is presented with the forceps.

The general initial steps of DE surgery also apply for bladder endometriosis (Figs 16 and 17). The procedure starts with the placement of a transurethral catheter. If cystoscopy is not performed the day before surgery, the catheterization is preceded by cystoscopy and the introduction of ureteral stents, when needed. Bladder endometriosis is usually easily identifiable: the bladder nodule may be immediately visible, or the round ligaments may be pulled medially, obliterating the ventral compartment due to fibrosis.

The dissection may start in the healthy peritoneum adjacent to the nodule, either paravesically or between the bladder and the uterus, depending on the size of the nodule or the adherence to the uterus or round ligaments. However, the tissue planes may sometimes be lost due to the disease, and the dissection may have to go through fibrotic tissue. As with hysterectomy, the dorsal wall of the bladder distally of the nodule needs to be found and dissected away from both uterus and the ventral wall of the vagina, thus opening the vesico- vaginal space. Special attention is needed if there is a history of Caesarean section. Finally, a soft plane of connective tissue is reached indicating the surgeon is distal of the nodule. The bladder is further mobilized coming from both sides to ensure subsequent suturing without tension. This step may be easier if the bladder is filled with at least 100 ml sodium chloride solution (and methylene blue) as its contours become more visible. When the nodule is isolated, and the bladder mobilized, the nodule is grasped with traction and excised with macroscopically free margins. It may be worth trying to resect the nodule from the detrusor muscle, respecting the mucosa if the latter is not involved, however this is possible only in the minority of cases. On the other hand, it is easier to control the dissection with an open bladder as the definition of the resection margins from both in- and outside is comfortable for the surgeon. Also, the trigone is directly visualized to avoid ureteric damage in this manner.

After resection, the bladder defect is closed horizontally with a running suture using 3-0 PDS, or another absorbable monofilament material. There is no evidence for whether a mono- or double layer suture results in a better outcome. If possible, only the detrusor is sutured and the mucosa avoided. After the suture, the leak tightness of the bladder is checked by filling it with 100 to 200 ml sodium chloride. Leakages are managed by single stitches. After large bladder resection the Retzius space may be opened to allow “a tension-free” bladder repair.

The complete resection of bladder lesions has its limitations when the uterus is involved and has to be preserved for fertility. A Robinson- or easy-flow- drainage can be considered postoperatively for the first 24-48 hours in order to have an early leakage detection.

In the case of bladder endometriosis close to the trigonum, ureteral stents introduced before or during the procedure facilitate the excision and closure procedure. A postoperative cystoscopy for inspection and assessment of the suture after 10 days of drainage is recommended by several authors (Rozsnyai et al., 2011; Antonelli, 2012, Maccagnano et al., 2012; Darwish et al., 2017; Leone Roberti Maggiore et al., 2017).

Traditionally, the transurethral resection with an electric loop of intra-vesical endometriosis is performed by some urologists. However, the disadvantage from our point of view is that only the tip of the iceberg is removed while too large a part of the nodule remains in situ. However, a cystoscopic approach may be combined with the laparoscopic route, during the same procedure, in large bladder nodules when limits are close to the ureter meatus in order to limit the risk of an inadvertent injury to the intravesical segment of the ureter.

##### Postoperative management

If there is significant postoperative haematuria, there may be a risk of catheter obstruction, and a transurethral rinsing catheter can be placed for continuous rinsing.

If an intraperitoneal drainage was placed during the operation, it should be removed on the first or second postoperative day.

The transurethral Foley catheter should remain in place for 8 to 10 days. Then, a radiological cystogram is highly recommended to check the integrity of the suture. If the suture is sufficient, the catheter can be removed.

Before discharging the patient from hospital, the amount of post-voiding urine is checked and an ultrasound of the kidneys is performed. Ureteral stents can be removed after approximately 6 weeks. Anticholinergic medication to reduce bladder irritability may be helpful while ureteric stents remain in place. The management described (Ulrich and Schüller, 2018) may especially be suitable for large resections. In patients with smaller defects after resection (for example when the lesions are more distant from the trigone or the ureteral ostia), the transurethral catheter can be removed earlier, and the ureteral stents can be removed immediately after suturing of the bladder, or not used at all.

##### Specific risks and complications of the resection of bladder endometriosis

A typical risk of partial bladder resection is secondary haemorrhage with bladder tamponade (i.e. large intravesical hematoma). This complication can usually be managed by continuous irrigation through a specific transurethral catheter, and a surgical revision is rarely required. Following large partial bladder resection, a reduced bladder capacity potentially aggravating the pre-existing problem may result.

In contrast to bowel surgery, insufficiency of the suture with postoperative leakage is less frequent in bladder surgery. This, however, may lead to urinoma formation requiring drainage and surgical revision, sometimes necessitating a long-lasting transurethral and/or suprapubic catheter (and rarely a nephrostoma). Postoperative hydronephrosis may occur by either accidental occlusion of the ureteral ostia (if the suture was performed close to the ureteral ostia), or lack of proper bladder emptying. This is usually prevented by the pre-operative placement of ureteral stents (double J). The development of fistulas is a complication of bladder and ureter surgery that can significantly affect the quality of life. It is more likely if concomitant complex urological, colorectal, and/or intestinal resections, or hysterectomies are performed. The use of an Omental flap can be considered in these cases.

#### Ureteral endometriosis

##### Presentation and symptoms

Ureteral endometriosis is often the result of pelvic sidewall endometriosis and is highly associated with DE of the uterosacral ligaments. If the ureter is affected by endometriotic implants, fibrosis, and inflammatory reactions that are attached to and consequently obstructing it from the outside, the situation is referred to as extrinsic ureter endometriosis. The infiltration of its muscular layer with or without reaching the lumen is defined as intrinsic ureteral endometriosis. Either form may cause hydronephrosis potentially leading to a complete loss of ipsilateral renal function. Unfortunately, hydronephrosis is often undetected due to non-specific or a lack of symptoms; only a minority of patients complain about colic-like pain or discomfort of the flank (mostly with a left predisposition) (Miranda-Mendoza et al., 2012). Hydronephrosis, due to endometriosis- associated ureteral obstruction, is considered an absolute indication for intervention if sufficient kidney function still exists. If kidney function is lost, however, nephrectomy is indicated. A multidisciplinary review with the involvement of a urologist is recommended.

##### Surgical treatment

###### 

**Figure 18:**
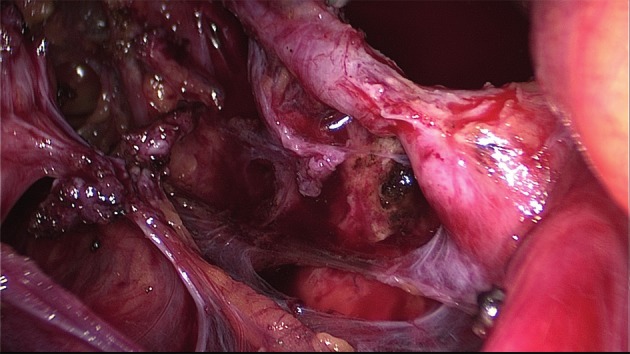
Intrinsic endometriosis in the left ureter with stenosis and hydronephrosis.

**Figure 19:**
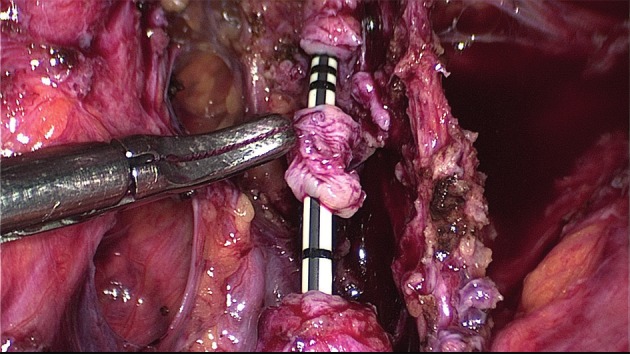
Segmental resection of the infiltrated part of the ureter.

**Figure 20:**
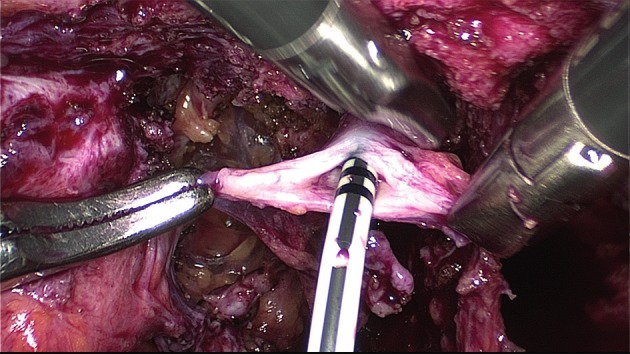
Incision of the distal stump of the ureter in order to increase the circumference in order to facilitate the end to end anastomosis and to decrease the risk of a post-operative stenosis formation.

**Figure 21:**
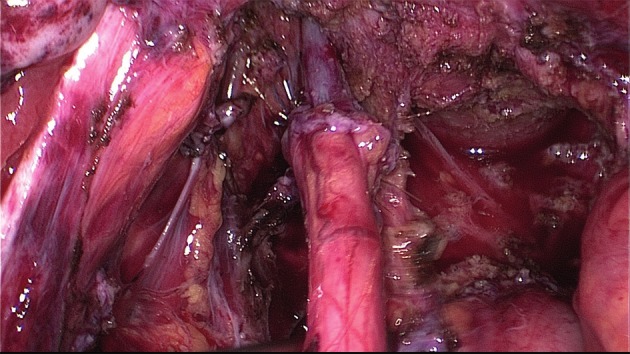
Final view of the end-to-end anastomosis (tension-free).

If ureteral endometriosis is already suspected pre-operatively, and/or if hydronephrosis is present, a pre-operative trial of ureteral stenting is recommended (Figs 18, 19, 20 and 21). During any ureteral intervention, care should be taken to avoid damaging the ureteral sheath within the vascular network (see the anatomy section).

###### Ureterolysis / Decompression

In cases of extrinsic ureteral endometriosis, ureterolysis is performed by freeing the ureter and excising the surrounding fibrotic tissue. This may also be successful in cases with the endometriosis reaching and infiltrating the adventitia– and even the muscular layer if no relevant obstruction of the ureter is present. Dissection of the ureter should be started in healthy tissue and not at the affected location.

###### Segmental ureteral resection (end-to-end anastomosis)

Segmental resection with end-to-end anastomosis may be indicated – provided that a tension-free anastomosis is possible – in cases after failed ureterolysis or when an intrinsic ureteral lesion distant from the bladder (i.e. in its upper sections) is present infiltrating the muscular layer with obstruction of the lumen. Technically, complete mobilization of the ends, spatulation, and suturing over an introduced ureteral stent are important for successful anastomosis. Interrupted sutures (e.g. 4-0 to 5-0, with monofilament material) are often used. The use of an omental flap in order to protect the suture has been recommended by some clinicians. In practice, this technique is feasible and safe when the length of the ureter involved by the stenosis does not exceed 1 cm: otherwise the tension-free anastomosis will not be achievable.

###### Ureteral re-implantation (ureteroneocystostomy)

Distal intrinsic lesions close to the bladder, stenosis with a length exceeding 1 cm, or failed ureterolysis (i.e. hydronephrosis has recurred or is still present after surgery) may require resection and re- implantation of the proximal end of the ureter into the bladder. Most often the psoas-hitch technique is preferred. As with ureterolysis, mobilization is started over the common iliac artery. Gentle dissection is continued all the way down to the affected portion of the ureter in order to protect the sheath with its blood supply. The ureter is transsected above the lesion. The distal -now blind- end is tied and will stay in situ. The bladder is mobilized entering the retropubic space (Retzius) thus creating a flap-like side so that the bladder and the proximal end of the ureter can be approximated. A cystotomy is performed at the bladder dome. An oblique tunnel is created by introducing a clamp into the bladder through that opening and advanced exactly to where the ureter will be implanted, thus creating another small opening, and the ureter will be pulled through that hole into the bladder. This is often done submucosally. The ureter is stented and its end spatulated. In the position described, the ureter is fixed to the bladder mucosa with 4-0 monofilament single stitches. Finally, the bladder is closed as previously described (see the bladder section) and a transurethral and/or supra-pubic catheter placed. The ureteral pig-tail stent(s) will be left in situ for approximately 6 weeks (Ceccaroni et al., 2019). Whether a psoas-hitch or a Boari procedure is the best way of performing the ureteral re-implantation (ureteroneocystostomy) may depend upon the situation and the urologist’s preference.

After ureteral surgery for both intrinsic- and extrinsic endometriosis, or if conservative treatment is preferred while DE is suspected, patients should be monitored by kidney ultrasound every 6 months in order to avoid overlooking silent hydronephrosis (Ulrich et al., 2014).

### DE of the diaphragm

#### Techniques used

Superficial lesions can be coagulated or ablated with different types of low-energy sources. The presence of larger lesions may afterwards be associated with diaphragmatic fenestrations, and therefore should be managed by a multidisciplinary team (Roman et al., 2008). It should be taken into account that only the ventral part of the diaphragm is visible by laparoscopy performed in dorsal lithotomy position. The left lateral decubitus position helps to have complete exposure of the right diaphragmatic muscle and endometriosis (Bourdel et al., 2019).

The diaphragmatic fenestrations are usually present in the tendinous (central) part of the diaphragm. These openings can lead to secondary pneumothorax, serous or bloody pleural effusions, or partial herniation of the liver into the chest.

In the case of catamenial pneumothorax, video- assisted thoracoscopic surgery (VATS) is the approach of choice for diagnosis and surgical treatment (Nezhat et al., 2007). In the case of lesions close to the phrenic nerve, a mini-thoracotomy can be considered (Alifano et al., 2007). Small fenestrations in the diaphragm can be closed with staples or interrupted stitches, while bigger defects should be excised and stitched. Especially in cases with large defects after resection, the thoracoscopic suturing by a thoracic surgeon is preferable. Plication or insertion of a mesh may be necessary in rare cases of larger diaphragmatic defects. “Blind” pleurodesis with talc without diagnosing diaphragmatic fenestrations can later lead to entrapped lung and isolated basal pneumothorax with persistent symptoms. Pleurodesis without excision of the endometriotic nodules is followed by continued pain during menstruation (Bagan et al., 2003). In a porous diaphragm, a polyglactin mesh implantation improves the outcome (Bagan et al., 2003). If deep lesions have been diagnosed without catamenial pneumothorax, a laparoscopic approach is a viable alternative. A surgeon with thoracic qualifications on standby is an essential prerequisite (Nezhat et al., 1998; Nezhat et al., 2007). Women who complain of catamenial pain in the (right) shoulder have diaphragmatic lesions until otherwise proven by thoracoscopy and laparoscopy performed in left lateral decubitus position. At thoracoscopy, only full thickness lesions are visible and some infiltrative lesions are not visible from the thoracoscopic side.

#### Risks and complications

The diaphragm is very thin and care should be taken to reduce the possibility of entering the thoracic cavity, particularly on the left side because of the proximity of the pericardium. Arrangements should be in place to manage a pneumothorax and other complications. Intra-operative placement of a pleural drain can solve the problem. Risks of VATS include postoperative pneumothorax, damage to the phrenic nerve, recurrences, postoperative haemothorax, and necessity for additional surgery due to concurrent pelvic endometriosis. The risks and complications of laparoscopy are identical in the case of additional VATS.

#### Prevention of complications

To limit the risk of pneumothorax, double lumen intubation is preferred over single lumen intubation (Alifano et al., 2007; Ciriaco et al., 2009). Intra- operative deflation after single lumen intubation has been used as an alternative; no comparative studies on this topic are available, however. A chest tube should be left in situ for 2-6 days. When surgery next to the phrenic nerve is necessary, a mini- thoracotomy can be helpful (Alifano et al., 2007; Roman et al., 2016b). Recurrences can be reduced through the use of a polyglactin mesh implantation and postoperative GnRH analogue treatment (Bagan et al., 2003; Ciriaco et al., 2009). If diaphragmatic endometriosis is found as the reason for catamenial pneumothorax, pelvic endometriosis must be investigated and excised.

When a patient does not want to have surgery or only incomplete resection is possible, in case of catamenial pneumothorax, a bilateral salpingo- oophorectomy may be considered if future fertility is not desired.

### DE of the abdominal wall including scars, the umbilicus, and the inguinal region

#### 

Endometriosis can be found in the abdominal wall, especially in scars, the umbilicus, or the inguinal region. These lesions may be misinterpreted as tumorous growths or keloids.

In abdominal scar endometriosis following laparotomy (fx. Caesarean section) or laparoscopy (fx. trocar insertion sites), the preferred method is wide excision.

A multidisciplinary approach may be required if a large defect needs to be repaired following excision. The use of a polypropylene mesh can be considered (Khan et al., 2017; Pas et al., 2017).

For umbilical endometriosis, a similar approach can be applied taking into account cosmetic consequences (Siddiqui et al., 2017).

In endometriosis of the inguinal region, the proximity to other structures (e.g. nerves and femoral vessels) should be considered and a multidisciplinary approach is advised.

#### Risks and complications

Incomplete excision and herniation through the fascia or rectus muscle have been reported. Women should be counselled about possible unavoidable and undesirable cosmetic consequences. Side effects from mesh cannot be excluded. Nerve damage and pain after resection of inguinal endometriosis is a risk.

### Frozen pelvis and endometriosis

A frozen pelvis is considered the ultimate stage of deep endometriotic lesions with fibrosis, severe adhesions, and abnormal tissue replacing pelvic soft tissue. It is defined as the presence of extensive dense adhesions at one or both adnexae with complete dorsal cul-de-sac obliteration. This definition may also include patients with severe peritoneal adhesions only.

The surgical management of these patients remains a challenge, as the risk of complications may be increased.

The basic principles of dissection are similar to those described for bowel endometriosis, even though there may not be true bowel endometriosis. For difficult cases and frozen pelvis, the general principles as described in Table II apply, in addition to the following principles:

- Try to mobilise the sigmoid colon.
- Identify the ureters and dissect if necessary.
- Follow the principles described in the specific chapters for the treatment of the uterosacral ligaments and rectovaginal septum, or urinary tract involvement.

### Hysterectomy in women with severe DE

#### 

Hysterectomy together with removal of endometriotic lesions, with or without oophorectomy, in women who have completed their family and/or have no desire to become pregnant, may be indicated in certain circumstances (Dunselman et al., 2014). Women should be carefully counselled about this irreversible decision to have a healthy organ removed, as well as the risks associated with a hysterectomy with oophorectomy, including a higher risk of coronary heart disease (Mu et al., 2016), congestive heart failure, obesity, and high blood pressure (Laughlin-Tommaso et al., 2018) as well as dementia (Rocca et al., 2012). Furthermore, they should be informed that hysterectomy will not necessarily cure the symptoms or the disease.

Hysterectomy for endometriosis is usually performed by the abdominal route (laparoscopy or laparotomy) depending on the extent of the disease. The laparoscopic route has the advantage of better access, identification, division of adhesions, and removal of non-uterine endometriotic lesions. It may also facilitate vaginal hysterectomy (LAVH) and ensure complete removal of all endometriotic lesions.

The general principles of laparoscopic hysterectomy should be applied in women with DE (Einarsson and Suzuki, 2009), with some specific recommendations.

#### Technical considerations

The principles of approach to deep endometriotic lesions should be followed, as described in the previous sections.

It is usually easier to leave the uterus until the endometriotic lesions are isolated or removed, but in certain situations performing hysterectomy first may facilitate access to the lesions and their excision. Sometimes an initial supracervical hysterectomy can precede the removal of the cervix in case of, for example, a large uterus, or to approach deep lesions. Treatment of the dorsal compartment and hysterectomy should be performed carefully. The plane used to excise the dorsal disease are the planes used in type B or C radical hysterectomy (Querleu et al., 2017) whereas in most patients without cardinal ligament involvement an intrafascial approach may be used to remove an adenomyotic uterus.

#### Ligation of the uterine arteries

In the case of an enlarged uterus or severe adhesions, consideration should be given to ligation of the uterine arteries at their origin as this may reduce bleeding and be helpful in the dissection of the ureter.

#### Treatment of rectal lesions

In the case of hysterectomy combined with resection of bowel endometriosis (anastomosis or full thickness resection), a temporary stoma can be considered.

#### Hysterectomy and bladder opening

In the case of the opening of the bladder or resection of a bladder nodule it is necessary to close the wound with a tension-free suture and avoid apposition of the bladder and vaginal vault by sufficient mobilisation of the bladder wall. The bladder should be drained with a catheter for 5 to 10 days.

#### Extraction of the uterus

If it is difficult to evacuate the uterus vaginally, morcellation in a bag can be considered, to prevent parasitic endometriosis.

#### Ureteral stenting

In the case of parametrial involvement at hysterectomy, the use of a stent may be helpful.

#### Caution of complications with hysterectomy in DE

Hysterectomy in the case of DE may be extremely challenging and is known to be associated with intra-operative complications (fourfold higher compared to normal hysterectomy): increased intra and postoperative risk of haemorrhage, and direct injuries (bowel, bladder, ureter) due to difficult adhesiolysis (Uccella et al., 2016; Surrey et al., 2017). It is particularly important to check the bowel and bladder integrity at the end of the procedure in difficult cases where extensive dissection is required. Adhesion prevention is to be considered in cases of extensive dissection; hyalobarrier gel and other anti-adhesion agents can be used.

## Conclusions

Surgery is an important treatment option for women with DE. However, like medical intervention, surgery is not always successful and is also associated with clinically relevant risks (Chapron et al., 1998; Becker et al., 2017). Surgical treatment failure can be partially attributed to the heterogeneity of endometriosis but it is also correlated with factors such as surgical experience, the complexity of each case, and anatomical locations of the disease.

The principles for identifying and treating deep endometriotic lesions (Table II) and the good practice recommendations in the text aim to support clinicians and surgeons is counselling and treating (or referring) women presenting with DE.

## 

**Supplementary Table SI:** — List of experts who contributed in the stakeholder review.

Reviewer Name	Organisation	Country
Martin Sillem	Scientific Endometriosis Foundation	Germany
Bulent Urman - Ercan Bastu, Ahmet Kale, Engin Oral, Yucel Karaman	Turkish Endometriosis and Adenomyosis Society	Turkey
Sukhbir Singh	CANADIAN SOCIETY FOR THE ADVANCEMENT OF GYNECOLOGIC EXCELLENCE, SOCIETY OF OBSTETRICIANS AND GYNECOLOGISTS OF CANADA	Canada
Edgardo D. Rolla	Sociedad Argentina de Endometriosis, San Isidro Medicina	Argentina
Deborah Bush	Endometriosis New Zealand	New Zealand
Reviewer Name		Country
Hélder Ferreira		Portugal
Emelie Faller		France
Bernhard Krämer		Germany
Alexander Ogurtsov		Russian Federation
Haytham Elmeligy		Germany
Jean Dubuisson		Switzerland
Raj H. Dodia		Kenya
Maribel De Gouveia De Sa		UK
Sven Becker		Germany
Popov A.		Russia
Dominic Byrne		UK
Nadya Magunska		Bulgaria
Chii-Ruey Tzeng		Taiwan
Marwa Fakhreldin		UK
Renato Seracchioli		Italy
Päivi Härkki		Finland
Ahmet Turp		Turkey
Tanya Timeva		Bulgaria
Xinmei Zhang		China
Melinda-Ildiko Mitranovici		Romania
Atanas Shterev		Bulgaria
Gernot Hudelist		Austria
Jim Tsaltas		Australia
Ioannis Gryparis		Greece
Carlos Calhaz-Jorge		Portugal
Santiago Díez		Spain
Karen Joseph		New Zealand
Mira Töyli		Finland
Tommaso Falcone		USA
Thomas Strowitzki		Germany
Miguel J. Flores Villalobos		Mexico
Jean Bouquet de la Jolinière		Switzerland
Carlos Alberto Petta		Brazil
